# Comparative *in silico* analysis of *ftsZ* gene from different bacteria reveals the preference for core set of codons in coding sequence structuring and secondary structural elements determination

**DOI:** 10.1371/journal.pone.0219231

**Published:** 2019-12-16

**Authors:** Ayon Pal, Barnan Kumar Saha, Jayanti Saha

**Affiliations:** Microbiology & Computational Biology Laboratory, Department of Botany, Raiganj University, Raiganj, West Bengal, India; UMR-S1134, INSERM, Université Paris Diderot, INTS, FRANCE

## Abstract

The deluge of sequence information in the recent times provide us with an excellent opportunity to compare organisms on a large genomic scale. In this study we have tried to decipher the variation in the gene organization and structuring of a vital bacterial gene called *ftsZ* which codes for an integral component of the bacterial cell division, the FtsZ protein. FtsZ is homologous to tubulin protein and has been found to be ubiquitous in eubacteria. FtsZ is showing increasing promise as a target for antibacterial drug discovery. Our study of *ftsZ* protein from 143 different bacterial species spanning a wider range of morphological and physiological type demonstrates that the *ftsZ* gene of about ninety three percent of the organisms show relatively biased codon usage profile and significant GC deviation from their genomic GC content. Comparative codon usage analysis of *ftsZ* and a core housekeeping gene *rpoB* demonstrated that codon usage pattern of *ftsZ* CDS is shaped by natural selection to a large extent and mimics that of a housekeeping gene. We have also detected a tendency among the different organisms to utilize a core set of codons in structuring the *ftsZ* coding sequence. We observed that the compositional frequency of the amino acid serine in the FtsZ protein appears to be a indicator of the bacterial lifestyle. Our meticulous analysis of the *ftsZ* gene linked with the corresponding FtsZ protein show that there is a bias towards the use of specific synonymous codons particularly in the helix and strand regions of the multi-domain FtsZ protein. Overall our findings suggest that in an indispensable and vital protein such as FtsZ, there is an inherent tendency to maintain form for optimized performance in spite of the extrinsic variability in coding features.

## Introduction

Codon usage bias (CUB) or the preference of an organism for a certain subset of codons coding for the different amino acids of polypeptides is an important evolutionary feature that has intrigued molecular biologists and evolutionists for decades [1,2]. It is a universal phenomenon observed in prokaryotes, eukaryotes [3] as well as viruses [4], and is predominantly dependent on selection, mutation, and genetic drift [5]. CUB exists as a balance between selective and neutral processes [6–10] and is shaped by several factors some of which include GC content, compositional bias, gene length, hydropathy, function, mutational bias and protein structure [11–15]. It has been found to be an important factor contributing to gene and genome evolution [15,16], and is a significant determinant of gene expression levels at the transcription level [3]. CUB has been reported to be responsible in controlling a variety of cellular processes such as translational efficiency, differential protein production and folding [17–19], and is a means to fine tune the expression of genes [17,20]. A strong CUB has been reported to be a characteristic feature of highly expressed genes [21–25]. The codon usage pattern of genes and genomes of a large number of organisms including bacteria, archaea, eukaryotes as well as viruses have been studied since the last five decades with the earliest record in PubMed [26] (https://www.ncbi.nlm.nih.gov/pubmed/) dating back to 1979 by Hasegawa et al. [27]. It has been found that codon usage pattern vary not only between genomes but also between coding sequences or genes within an organism [5,6]. In this study we have tried to decipher the variation in the gene organization and structuring of a vital bacterial gene called *ftsZ* which codes for an integral component of the bacterial cell division, the FtsZ protein. The process of bacterial cytokinesis is initiated by the assembly of the tubulin-like GTPase called FtsZ which is essential for bacterial cell division [28]. During bacterial cytokinesis, the FtsZ protein is recruited initially at the future division site which then polymerizes itself into a ring like structure called the Z ring [29,30] consisting of short, 30 subunits long, FtsZ protofilaments [31–33]. The Z ring provides a stage for assembly of the cell division apparatus and constricts at the leading edge of the invaginating septum [28]. The Z ring appears as a smooth, closed circular assembly under fluorescence light microscope [30,34]. The polymerization of FtsZ is dependent on GTP hydrolysis [35] and the remaining GDP bound FtsZ favours depolymerisation [36]. FtsZ is homologous to tubulin protein which acts as the building block of the microtubule cytoskeleton in eukaryotes FtsZ [37]. During cell division, FtsZ interacts with other membrane associated proteins like FtsW, FtsK, FtsQ and FtsI and helps in anchoring FtsZ to the bacterial cytoplasmic membrane [38]. FtsZ is reported to be a highly conserved protein [37] with a relative molecular mass of 40,000 and is ubiquitous in eubacteria. It is also found in the members of Euryarchaea, chloroplasts of plants and some mitochondria [39]. Higher plants have also been found to contain two distinct families of FtsZ homologues that seem to have diverged early in the evolution of plants [40]. Mutant bacteria which lacks FtsZ protein cannot divide but elongate into filamentous form. FtsZ is a vital cell-division protein in prokaryotes and is showing increasing promise as a target for antibacterial drug discovery [41]. The FtsZ protein has been projected as a potent target and has been studied extensively [42] for the discovery of next-generation antibacterial agents that can be used to counter drug-resistances to the commonly used drugs for methicillin resistant *Staphylococcus aureus* (MRSA), tuberculosis, and other microorganism mediated infections [43]. The *ftsZ* gene is regarded as an essential cell division gene in many bacteria including *E*. *coli* [44] and it has been found that the C-terminal domain for FtsZ is highly variable both in size and alignment among the different bacterial species [45]. The higher-order structure of FtsZ protein *in vitro* includes ribbons, sheets and bundles [46–48].

Considering the critical nature and ubiquity of the FtsZ protein encoded by the *ftsZ* gene, it presents a fitting proposition to decipher the codon usage pattern of the *ftsZ* gene across the entire eubacterial domain to find out if there exist any differential codon usage bias in the structuring of the *ftsZ* coding sequence among different types of bacteria. The conservedness, ubiquity and imperative nature of the FtsZ protein in a critical bacterial cellular process like cytokinesis also portrays the *ftsZ* gene as a perfect candidate for housekeeping gene. The term ‘housekeeping genes’ refer to those genes that are required for the upkeep of basic cellular processes for the survival of a cell [49,50]. In this study, we have extensively analysed the codon usage pattern of the *ftsZ* gene from a wide range of bacteria differing in terms of their lifestyle and Gram nature, and spread across about 70 families belonging to 40 orders under 20 different classes encompassing the entire eubacterial domain to detect the factors shaping codon usage bias in *ftsZ*, and find out if the codon usage pattern of the *ftsZ* gene within different types of bacteria is a random phenomenon or has it been influenced by traits such as the lifestyle of the organism [51–55]. This includes their free-living behaviour or pathogenic association with specific host organisms and ecological traits. We have also tried to unravel whether the codon structuring of the *ftsZ* gene is influenced by the Gram nature of the organism to a certain extent. The Gram nature of a bacterium, although primarily attributed to the cell wall construction of the bacterium, have been found to manifest a host of comparative features in the organisms ranging from simple morphology [56] to advanced physiological, biochemical, ecological [57–59] and molecular characteristics such as GC content [60,61]. We have also compared the codon usage pattern of the *ftsZ* gene with a known bacterial housekeeping gene *rpoB*, coding for the β subunit of the bacterial RNA polymerase, to examine to what extent the codon usage pattern of the *ftsZ* gene deviate or resemble that of a core and conserved housekeeping gene such as *rpoB*. We have also attempted to estimate the compositional divergence of the *ftsZ* coding sequences. The FtsZ protein is a very vital component of bacterial cell division that demonstrates promiscuous variability both in terms of gene sequence and amino acid composition. This compositional variability in a conserved protein such as FtsZ has been our impetus to decipher and track whether there exists the preference for a ‘core’ set of codons in coding the gene sequence across a diverse group of bacteria. In our study we have tried to explore the codon usage tendency based on the positioning of the different amino acids in the different types of structural elements of the FtsZ protein. It has been reported that codon usage can play an important role in the translation process as well as the folding behaviour of nascent polypeptides [62,63]. We have adopted a unique approach to further explore the codon usage bias profile of the *ftsZ* sequence by linking the codon utilization profile with the secondary structural components of the protein. Thus, we have strived to correlate the coding pattern of the *ftsZ* gene with the structural attributes of the FtsZ protein. We have meticulously analysed the 61 sense codons coding for the twenty standard amino acids to find out the preference of disposition of specific codons in specific secondary structural elements of the FtsZ protein.

## Materials and methods

The *ftsZ* and *rpoB* CDS of 143 bacterial species spanning the entire eubacterial domain and their whole genome sequences were retrieved from the NCBI GenBank [64] sequence database. The *ftsZ* and *rpoB* coding sequences (CDS), and their corresponding amino acid sequences were extracted from the whole genome sequences of the bacteria. Details regarding the NCBI accession number of the genomes, links pertaining to the genome information page, reference to the information regarding the organism in the corresponding volumes of Bergey’s Manual of Systematic Bacteriology [65–69], and locus tag/protein id of the *ftsZ* and *rpoB* CDS is provided in S1 Table. Analysis of the different codon usage bias parameters like effective number of codons (Nc) [70], GC content, guanine and cytosine content at the first (GC1), second (GC2) and third position of the codon (GC3) [70] and hydrophobicity were also estimated using CodonW [71] and INCA 2.1 [72].

The Nc determines the degree of bias for the use of codons [2] with value ranging from 20 to 61, where lower value indicates higher codon usage bias and vice versa. It is a commonly used measure to quantify how far a gene departs from the equal usage of synonymous codons [73,74]. The GC content plays a critical role in genome evolution [75], and it has been found to range from 13% to 75% in cellular organisms [76,77]. The GC content does not remain constant throughout the genome of an organism but varies based on different regions and coding sequences of the genome. The measurement of different GC based attributes like GC content, GC1, GC2 and GC3 content thus play a significant role in analysing the genomic as well as genic organization [18,78]. The percentage of genes in the genome of an organism with Nc and GC3 less than that of both *ftsZ* and *rpoB* along with their standard score (*z*-score) [79,80] was also calculated to determine how far the codon usage bias of *ftsZ* and *rpoB* differ with respect to each other, and the genome of the individual organism. The standard score (*z*) of a raw score (*x;* where *x* could be Nc/GC3 score of a CDS) was calculated as *z* = (*x*-*μ*)/*δ* where *μ* is the mean of the population (or genome) and *δ* is the standard deviation of the population (or genome of a bacterium). The Nc–plot [70], which is a parabolic curve used to measure and explore codon usage bias, and detect the effect of base content on CUB [81] was constructed using Microsoft Office Excel 2013 version.

Statistical analysis such as non-parametric One way ANOVA on Ranks [82] was used to find out whether there is a preferred set of codon for each of the amino acid that is used in the structuring of the *ftsZ* coding sequences. Two factor ANOVA on codon usage of *ftsZ* CDS was also performed to study the frequency of the individual 61 sense codons and their interrelation with lifestyle and Gram nature of the organisms. Furthermore, a two factor ANOVA was designed to study the interrelationship of the twenty different amino acids with lifestyle and Gram nature of the bacteria. Data analysis pack of Microsoft Office Excel 2013 version was used to perform all the statistical analyses.

The degree of identity in FtsZ protein sequences among the 142 organisms considered for this study was analysed using the Windows 64-bit version of Clustal Omega 1.2.2. This application employs HMM profile-profile techniques along with seeded guide trees to produce multiple alignments [83]. For clustering of similar proteins based on their sequence similarities, the web server version of the program CD-HIT [84] (http://weizhong-lab.ucsd.edu/cdhit-web-server/cgi-bin/index.cgi) was used. Multiple sequence alignment (MSA) of the 142 *ftsZ* CDS was also constructed using Clustal Omega [83] and a phylogenetic tree depicting the relationship between all the considered *ftsZ* CDS was generated using the Molecular Evolutionary Genetics Analysis or MEGA X software graphical interface based 64-bit version for Windows [85]. The phylogenetic tree was inferred using the neighbour-joining method [86] with 1000 bootstrap replicates [87], and the evolutionary distances, measured in terms of number of base substitutions per site, were computed using the Tajima-Nei method [88]. The rate variation among sites was modeled with a gamma distribution. Visualization of the phylogenetic tree was carried out using Interactive Tree of Life (iTOL) ver. 4.4.2, a web based tool hosted at https://itol.embl.de/ [89,90]. The MSA data in FASTA format and the raw phylogenetic inference data along with bootstrap support in Newick format is provided in the supplementary files S1 MSA and S1 Phylogeny respectively. All the 143 *ftsZ* CDS were further subjected to clustering using CD-HIT with a similarity threshold of 50%.

Representative amino acid sequences of *ftsZ* of the four main clusters as identified by CD-HIT were subjected to secondary structure (helix, strands and other elements) prediction using the SSpro 5.2 module of SCRATCH Protein Predictor (http://scratch.proteomics.ics.uci.edu) [91]. Accurately predicting protein secondary structure is important for the study of protein evolution, structure and function. The *ftsZ* CDS were further aligned with their corresponding amino acid sequences and secondary structure mark-up sequence was generated using SSpro 5.2. With the help of this triple alignment, we have identified each of the synonymous codons that are used for coding the amino acids, and we have linked those codons with the amino acids of the predicted secondary structural elements (SSE). The relative synonymous codon usage (RSCU) value which is measured by the ratio between the actual observed values of the codon and the theoretical expectations was also calculated. RSCU reflects the relative usage preference for the specific codons encoding the same amino acid [92]. If RSCU value equals to 1, codon usage is supposed to be unbiased but if RSCU>1, specific codon frequency is higher than other synonymous codons and codon usage is considered to be biased [93]. The RSCU values of the *ftsZ* CDS were calculated after splitting the sequences based on their propensity in constituting the different secondary structural classes as predicted by SSpro 5.2 [91].

## Results and discussion

A comprehensive codon usage analysis of the *ftsZ* gene and its corresponding protein (FtsZ), along with the housekeeping gene *rpoB* was carried out in 143 spp. of bacteria of which 75 are non-pathogenic and 68 are pathogenic in nature. On the basis of the nature of cell wall, 43 are Gram positive, 99 organisms are Gram negative and one organism called *Gardnerella vaginalis* 409–05 is Gram variable in nature. A list of the organisms considered in this study along with their Gram nature and lifestyle is given in the file S2 Table.

After analysing the codon usage data of *ftsZ* given in S2 Table from the 142 species we found that *Kocuria kristinae*, which is a pathogenic, Gram-positive bacteria exhibits the lowest Nc value (24.56) among all the organisms. On the other hand, *Chlamydophila pneumoniae* CWL 029, a pathogenic, Gram-negative bacteria exhibited the highest Nc value of 61. A higher Nc value indicates poor codon bias of the gene [94]. Analysing the mean genomic Nc value of all the organisms studied, it was observed that the lowest mean genomic Nc value (29.198) is depicted by the organism *Kocuria kristinae*, a pathogenic, Gram-positive bacteria whereas the maximum mean genomic Nc value (55.68) is depicted by a pathogenic, Gram-negative bacteria called *Anaplasma marginale* str. Florida. Our observations primarily suggest that the degree of codon bias in the pathogenic organisms span a wider range. Analysis of the codon usage pattern of the *rpoB* gene (S3 Table) demonstrated *Kocuria kristinae* to exhibit the lowest Nc value (25.39) which is in line with its *ftsZ* Nc value.

Following the trend in Nc values, we clearly observed that the mean genomic Nc in majority of the organisms is higher than the genic Nc of *ftsZ* as well as *rpoB*. This suggests that the *ftsZ* gene is subjected to greater codon bias in comparison to the genomic Nc, a trend resembling the housekeeping gene *rpoB*. But in case of the *ftsZ* CDS of eight organisms, exceptions were evident where the genic Nc of *ftsZ* was found to be greater than that of the mean genomic Nc. These organisms include *Haemophilus influenzae* Rd KW20, *Halomonas boliviensis* LC1, *Geobacillus subterraneus*, *Anaplasma marginale* str. Florida, *Coxiella burnetii* RSA 493, *Caldicellulosiruptor bescii* DSM 6725, *Brucella melitensis* bv. 1 str. 16M and *Chlamydophila pneumoniae* CWL029. Most of these organisms are Gram negative and pathogenic in nature. When compared to *ftsZ*, the Nc score of *rpoB* was however found to lesser than that of *ftsZ* in a majority of the bacterial species including the eight species mentioned above suggesting comparatively greater codon bias in the housekeeping gene. About 30% of the 143 bacterial species considered in this study demonstrated lower Nc values for *ftsZ* in comparison to *rpoB*. The pathogenic bacterial species such as *Corynebacterium diphtheria*, *Chlamydophila pneumoniae* CWL029 and *Brucella melitensis* bv. 1 str. 16M demonstrated *ftsZ* Nc much higher than that of *rpoB*. A large number of Gram negative bacterial species were found to display genic Nc of *ftsZ* lower than that of *rpoB*. These organisms include *Treponema denticola* ATCC 35405, *Porphyromonas gingivalis* ATCC 33277, *Geobacter sulfurreducens* PCA, *Francisella philomiragia* subsp. philomiragia ATCC 25017, *Eikenella corrodens* ATCC 23834, *Salinibacter ruber* DSM 13855, *Prochlorococcus marinus* str. AS9601, *Xylella fastidiosa* 9a5c, etc.

**Fig 1:**
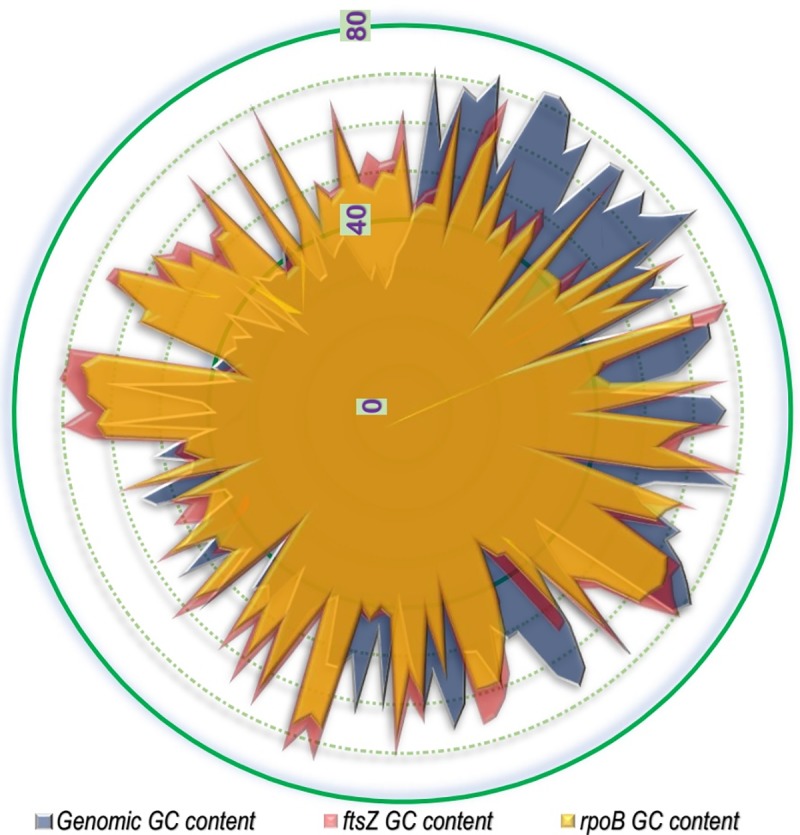
A superimposed radar plot comparing the genomic GC content with that of the *ftsZ* and *rpoB* CDS GC content of the 143 bacterial species considered in this study. From the centre to the periphery each concentric ring represent increase in 10% GC content.

A comparative study of the genomic GC content with that of the *ftsZ* and *rpoB* genic GC content illustrated in Fig 1 clearly demonstrates that in a significant majority of the bacterial species the genic GC content of *ftsZ* CDS is greater than that of *rpoB* CDS. In comparison to the genomic GC content, nearly 54% of the studied bacterial species were found to have genic *ftsZ* GC content greater than the genomic GC content, whereas in the case of *rpoB*, about 47% of the organisms were found to have a genic GC content greater than that of the genomic one. In terms of GC3 content, *Arcobacter butzleri* RM 4018, a pathogenic Gram negative strain depicted the lowest GC3 value for *ftsZ* gene (0.01974). The maximum GC3 content for *ftsZ* was shown by *Methylobacterium aquaticum* (0.9819), a non-pathogenic Gram negative bacteria. When compared to *rpoB*, the GC3 of nearly 40% bacterial species considered in this study was found to be less than that of *ftsZ*, and majority of these are non-pathogenic in nature.

The study of the relation between Nc and GC3 is an important analytical tool for examining codon bias. In order to explore the relationship between the Nc and GC3 attributes of both the *ftsZ* and *rpoB* CDS, a Spearman’s rank correlation analysis was performed. This detected a negative correlation between the Nc and GC3 of *ftsZ* (*ρ* = -0.491, p<0.01) as well as *rpoB* (*ρ* = -0.562, p<0.01). To further clarify the degree of association between Nc and GC3 of *ftsZ* and *rpoB* CDS, non-linear regression analysis was carried out and we observed that a polynomial cubic equation was best fit to describe the relationship between Nc and GC3 (*R*^2^ = 0.71; standard error of estimate = 3.72) compared to a linear (*R*^2^ = 0.18; standard error of estimate = 6.22) or a polynomial quadratic (*R*^2^ = 0.69; standard error of estimate = 3.79) equation. A similar trend was also observed in case of *rpoB* where a polynomial cubic equation was best fit to describe the relationship between Nc and GC3 (*R*^2^ = 0.63; standard error of estimate = 3.78). The *R*^2^ value of *ftsZ* was found to greater than that of *rpoB* suggesting a relatively stronger association between Nc and GC3 of the *ftsZ* CDS in comparison to *rpoB*.

**Fig 2:**
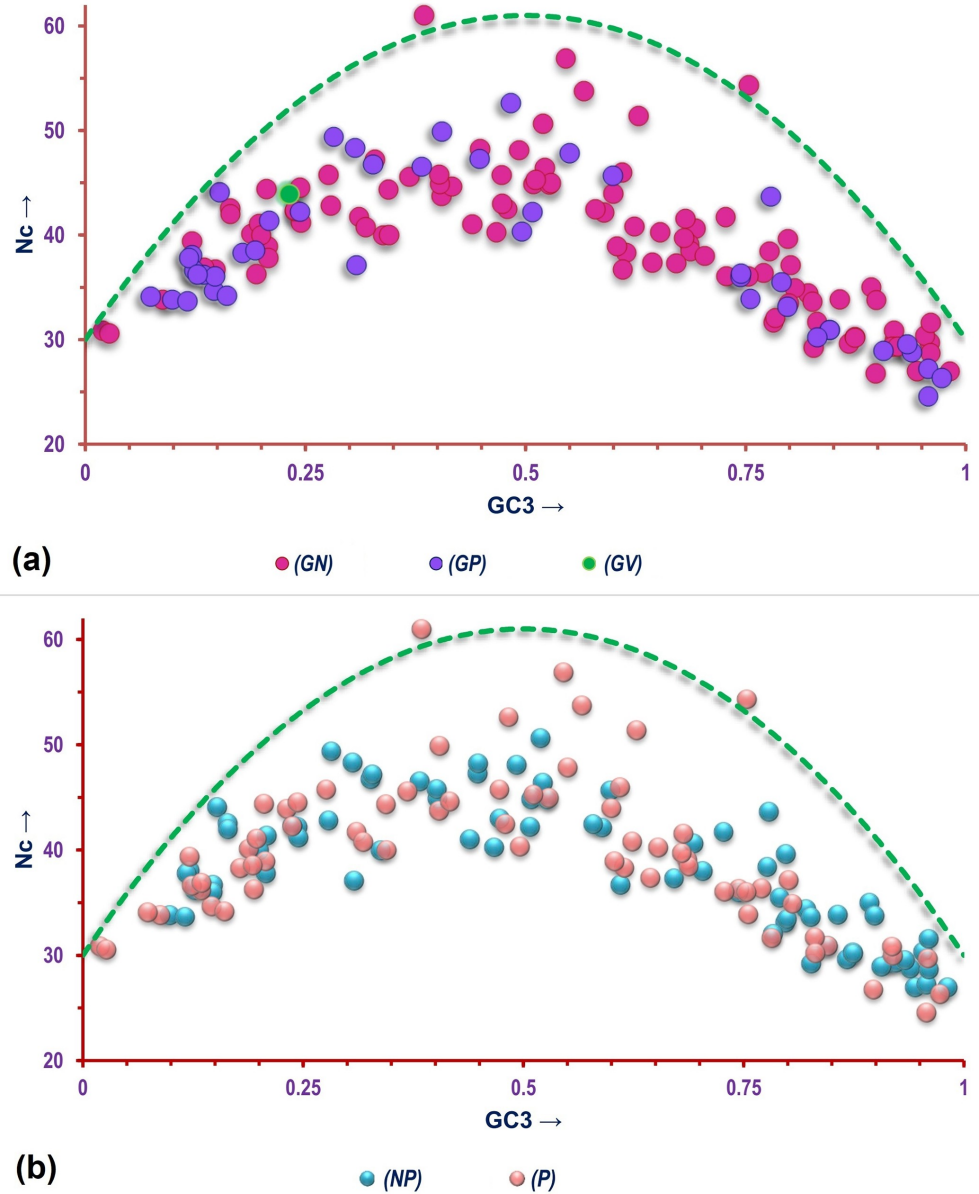
Nc-plots depicting the correlation between Nc and GC3 of the 143 *ftsZ* CDS selected for this study based on Gram nature and lifestyle. (a) Nc-plot constructed by demarcating the *ftsZ* CDS on the basis of Gram nature where GP = CDS of Gram positive bacterial species, GN = CDS of Gram negative species and GV = CDS of Gram variable bacteria. (b) Nc-plot constructed by demarcating the *ftsZ* CDS on the basis of lifestyle where NP = CDS of non-pathogenic and P = CDS of pathogenic bacterial species. The green dashed curve in both (a) and (b) depicts the null hypothesis that the GC bias at the synonymous site is solely due to mutation but not selection.

**Fig 3:**
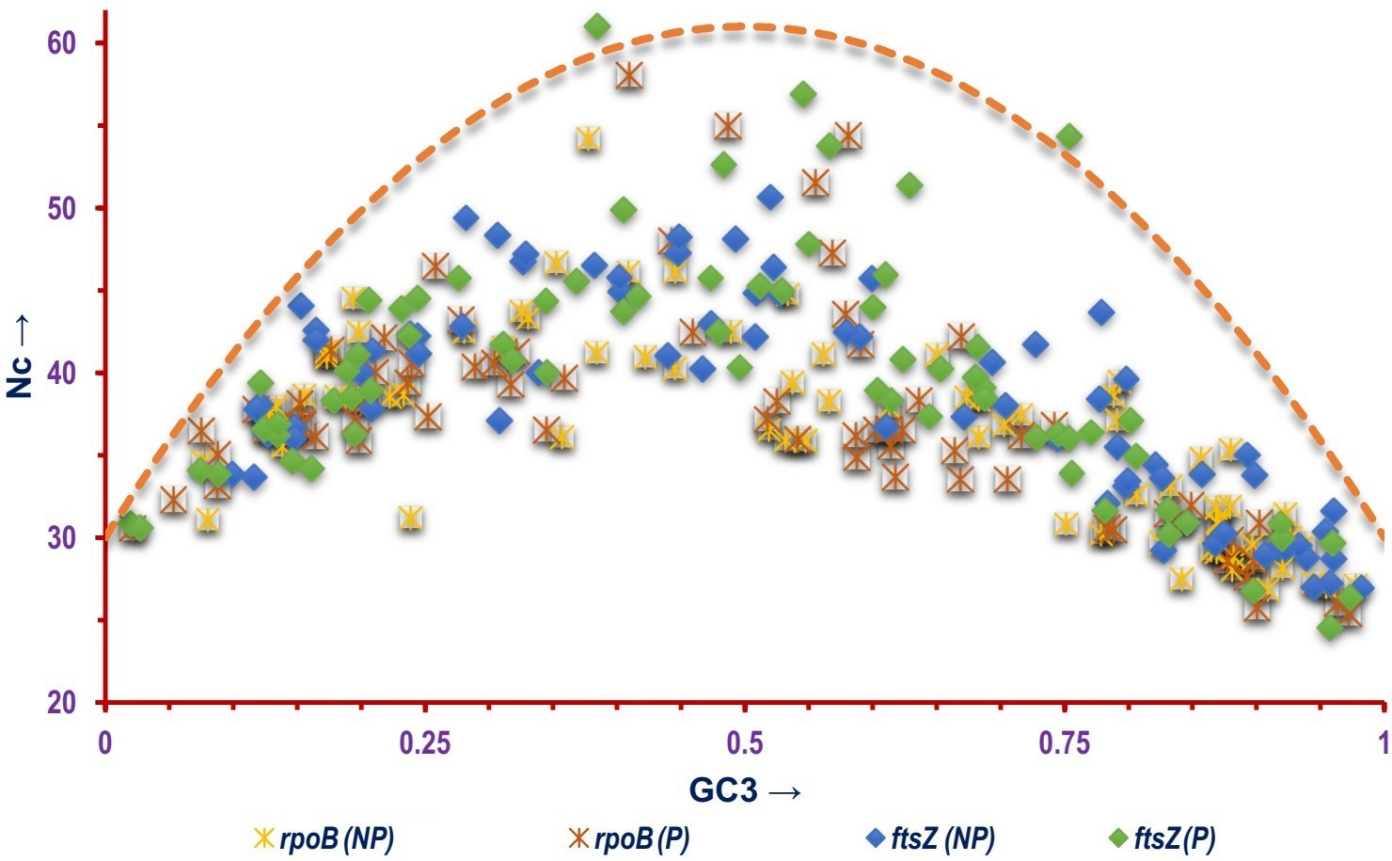
A composite Nc-plot depicting the correlation between Nc and GC3 of the *ftsZ* and *rpoB* CDS selected for this study based on lifestyle of the bacterial species where NP = CDS of non-pathogenic and P = CDS of pathogenic bacterial species. The orange dashed curve represents the null hypothesis that the GC bias at the synonymous site is solely due to mutation but not selection.

In order to better comprehend the codon usage bias profile of the *ftsZ* genes Nc-plots were constructed, delineating the *ftsZ* gene sequences based on the Gram nature and lifestyle of the organisms (Fig 2). Analysis of Fig 2(A) clearly demonstrates the *ftsZ* genes of majority of Gram positive bacterial species to lie well below the null hypothesis curve compared to the Gram negative ones. In terms of lifestyle, the *ftsZ* gene sequences of the non-pathogenic organisms were all found to scatter below the null hypothesis curve on the Nc-plot shown in Fig 2(B), suggesting translational selection as a major mechanistic force in shaping codon usage pattern in these bacterial species [70]. Analysis of the Nc-plot shows that the *ftsZ* genes of three pathogenic organisms—*Anaplasma marginale* str. Florida, *Brucella melitensis* bv. 1 str. 16M and *Chlamydophila pneumoniae* CWL029 occupy distinct positions on the Nc-plot (Fig 2). The common features shared by these three organisms are that they are Gram negative and pathogenic in nature. The bacteria *Anaplasma marginale* is a member of the order Rickettsiales. It is a small, obligate intracellular bacteria that typically have short genomes due to reductive evolution and survive as endosymbionts. It is also responsible for an infectious, non-contagious disease called bovine anaplasmosis in cattle and other ruminants [95]. The other organism *Brucella melitensis* is responsible for brucellosis, a common health hazard in people living in close vicinity of cattle [96]. The third organism called *Chlamydophila pneumoniae* represents an intracellular pathogen instigating different acute and chronic infections and has been found to be associated with chronic neurological disorders such as Alzheimer's disease and multiple sclerosis. Infection by *C*. *pneumoniae* which is a common cause of human respiratory disease [97] has also been suspected to cause chronic fatigue syndrome and the linked syndrome polymyalgia rheumatic in some patients [98]. A composite Nc-plot shown in Fig 3, consisting of both the *ftsZ* and *rpoB* CDS clearly depicts all the *rpoB* sequences to lie well below the null hypothesis curve suggesting selectional pressure as a major determinant of CUB [70]. In case of both *rpoB* and *ftsZ*, we observed that the gene sequences of some of the pathogenic species were found to be located much closer to the null hypothesis curve.

In order to study the compositional divergence of the gene sequences coding for FtsZ protein in the selected organisms with respect to their whole genome, the difference between the mean genomic Nc with Nc of *ftsZ*, the difference between average whole genome GC3 content with that of the *ftsZ* CDS and genomic GC content with the GC content of *ftsZ* gene was analysed. The relationship between GC1, GC2 and GC3 was also analysed to have a better understanding of the forces shaping codon usage pattern in both the *rpoB* and *ftsZ* gene sequences.

### Comparative analysis of mean genomic Nc and *ftsZ* Nc

**Fig 4:**
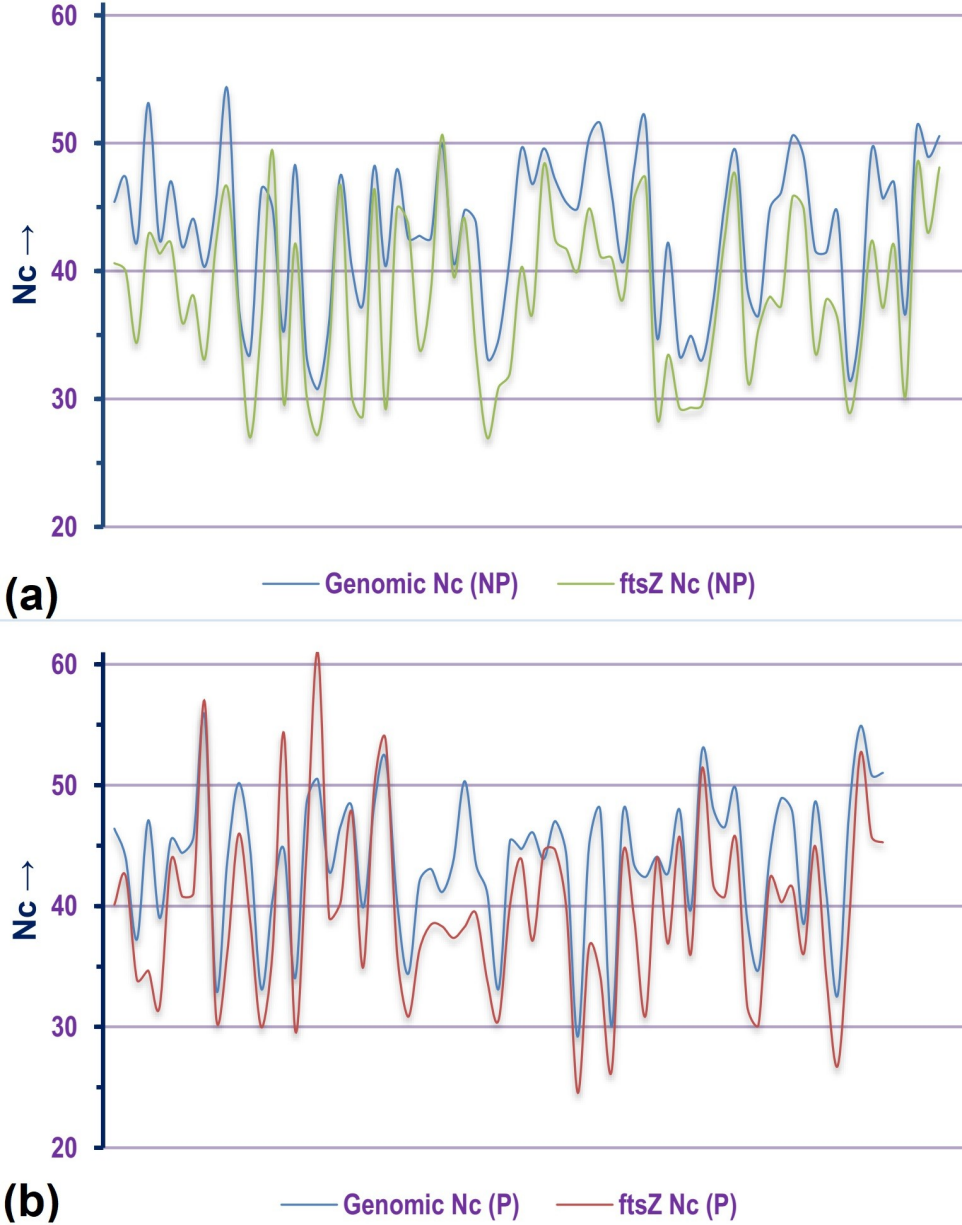
A comparative graphical representation of the genomic Nc and Nc of *ftsZ* CDS from (a) non-pathogenic [NP] bacterial species and (b) pathogenic [P] bacterial species included in this study.

**Fig 5:**
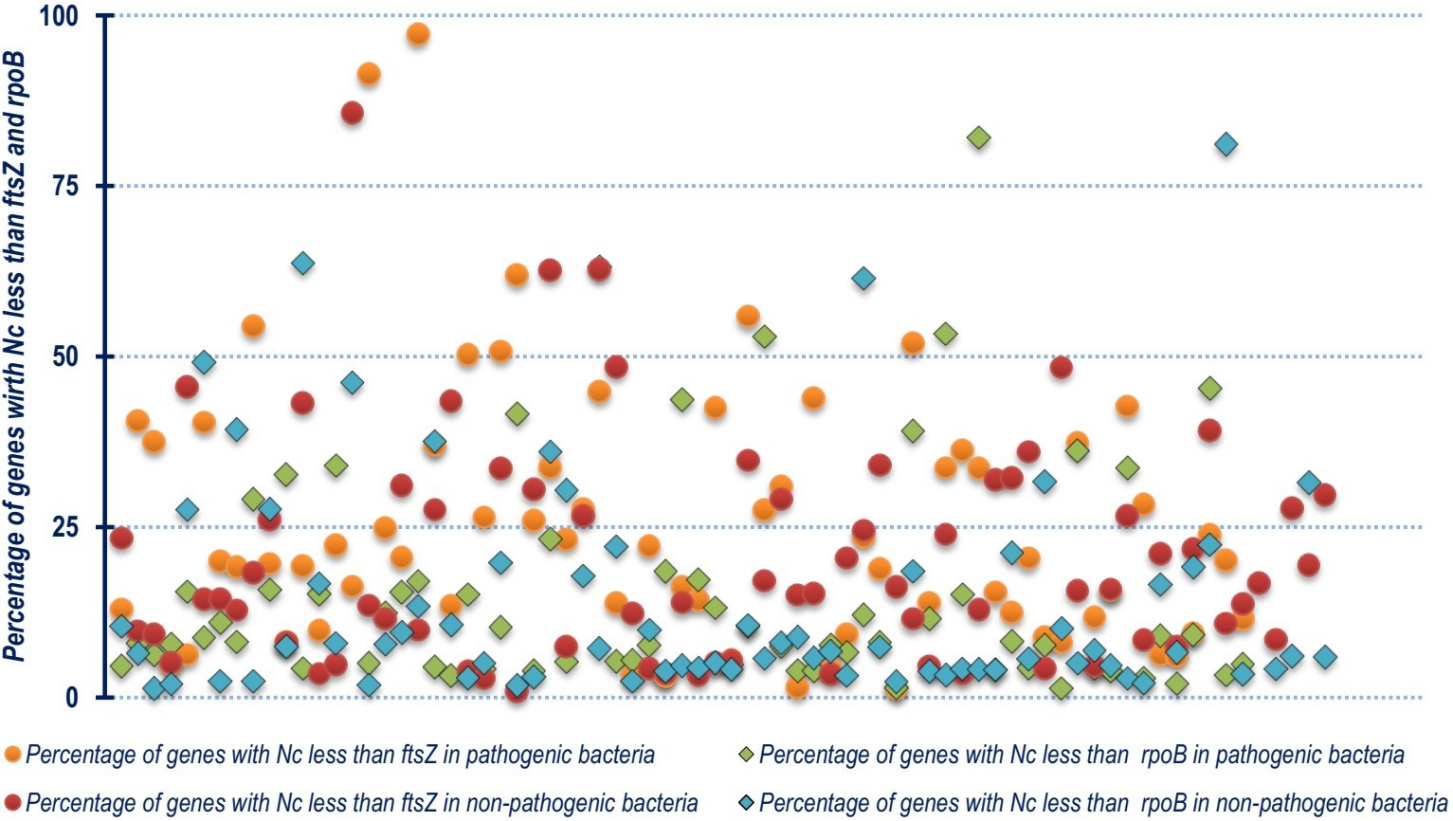
A scatter plot showing the distribution of the percentage of genes with Nc less than that of *ftsZ* and *rpoB* residing within the genome of each of the organism segregated on the basis of the non-pathogenic and pathogenic nature of the organism.

**Fig 6:**
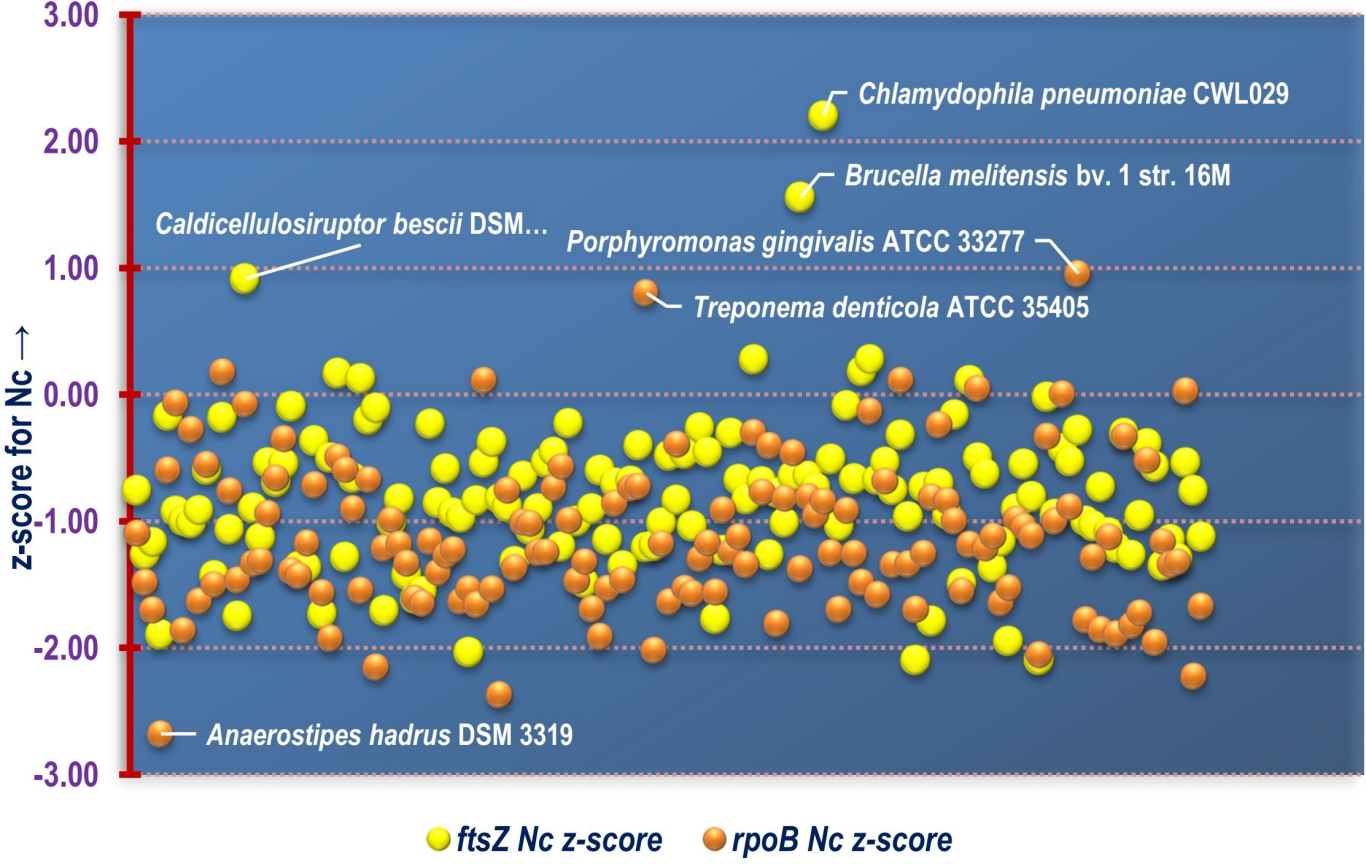
A scatter plot depicting the distribution of z-score for the Nc of *ftsZ* and *rpoB* CDS of all the bacterial species included in this study. The name of the organisms with relatively higher and lower Nc z-score have been labelled in the plot.

Out of the 143 organisms, about 93% (134 species) demonstrated relatively biased codon usage configuration in terms of Nc value. Out of these, 21 species *viz*., *Lactococcus garvieae* Lg2, *Ensifer adhaerens*, *Mycobacterium abscessus*, *Aerococcus viridans*, *Cupriavidus metallidurans* CH34, *Enterococcus avium* ATCC 14025, *Deinococcus radiodurans* R1, *Lactococcus lactis* subsp. lactis Il1403, *Butyrivibrio proteoclasticus* B316, *Neorhizobium galegae* bv. orientalis str. HAMBI 540, *Ochrobactrum anthropi* ATCC 49188, *Geobacter sulfurreducens* PCA, *Rhizobium etli* CFN 42, *Polynucleobacter asymbioticus* QLW-P1DMWA-1, *Burkholderia ubonensis* MSMB22, *Granulibacter bethesdensis* CGDNIH1, *Alteromonas macleodii* ATCC 27126, *Sinorhizobium fredii* NGR234, *Serratia fonticola* and *Streptococcus pneumoniae* R6 demonstrated Nc values of *ftsZ* CDS that are 20% or less than their mean genomic Nc values. This is suggestive of a significant codon bias existing within the *ftsZ* CDS. On the other hand, the *ftsZ* CDS of two Gram negative and pathogenic species *Chlamydophila pneumoniae* CWL029 and *Brucella melitensis* bv. 1 str. 16M were found to display Nc values twenty units greater than their mean genomic Nc score. A comparative graphical representation of the genomic Nc and *ftsZ* Nc segregated on the basis of lifestyle is depicted in Fig 4. The percentage of genes with Nc less than that of *ftsZ* residing within the genome of each of the organism was also calculated (S5 Table) and compared with that of *rpoB*. From Fig 5 it is clearly evident that majority of the species considered in this study have relatively fewer percentage of genes with Nc score below that of *ftsZ* as well as *rpoB*, which is a core bacterial housekeeping gene. A common trend was visible in the non-pathogenic bacterial species where a majority depicted relatively fewer number of genes with Nc score below that of *ftsZ* and *rpoB*. Furthermore, the analysis of z-score as a relative statistical measure to normalize and compare the difference between mean genomic Nc with *ftsZ* and *rpoB* genic Nc (S6 Table) in the 143 bacterial species, shown in Fig 6, demonstrates that a significantly large number of bacterial species have *ftsZ* and *rpoB* genic Nc lower than that of the mean genomic Nc. This is a significant indicator of greater codon bias existing within the CDS of *ftsZ*, and a trend well in line expected of a housekeeping gene like *rpoB*. Organisms with exceptional Nc z-score for *ftsZ* and *rpoB* CDS indicating significantly reduced codon bias compared to the genome have been pointed out in Fig 6.

### Comparative analysis of genomic GC3 and genic *ftsZ* GC3

**Fig 7:**
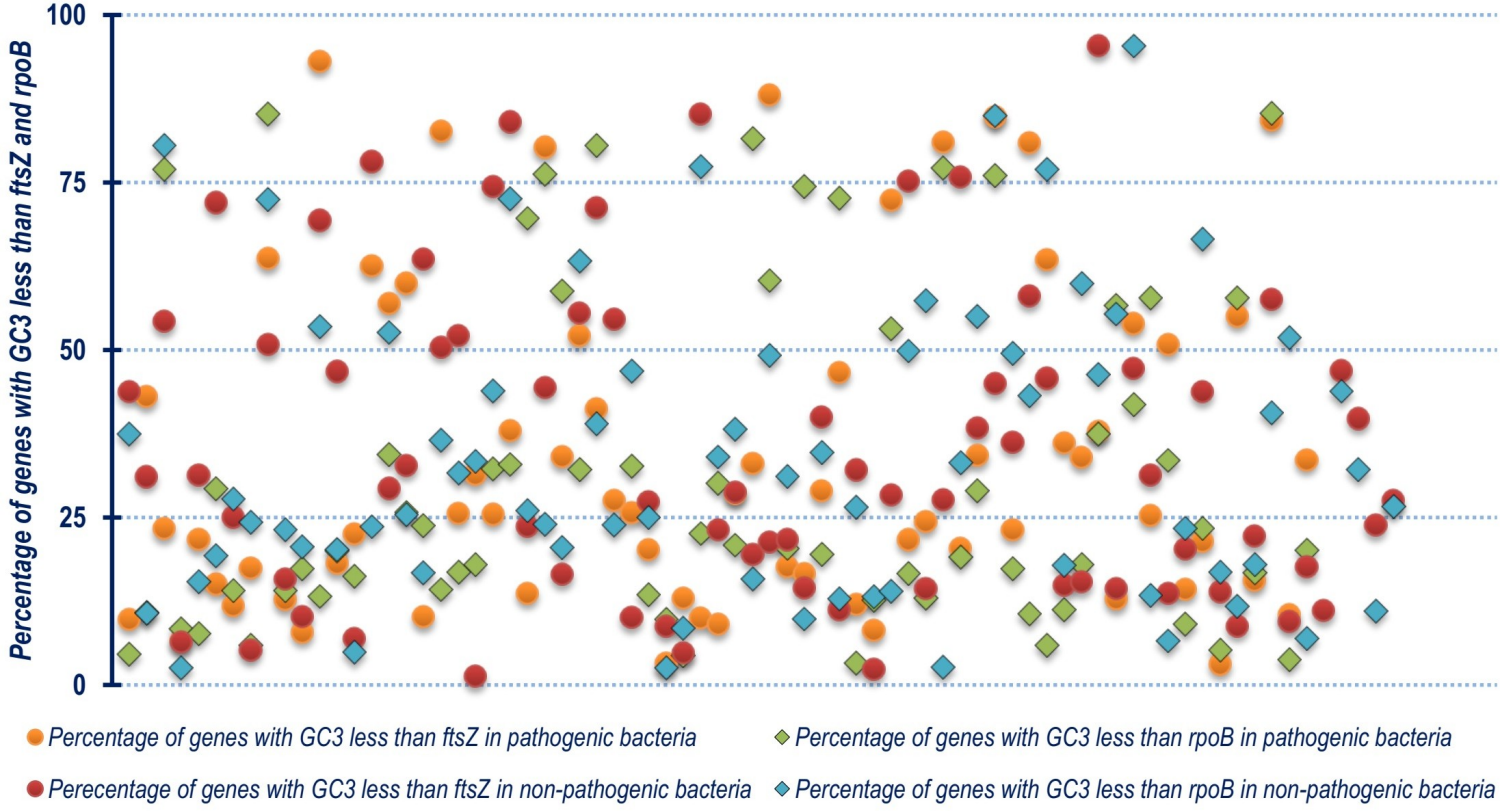
A scatter plot showing the distribution of the percentage of genes with GC3 less than that of *ftsZ* and *rpoB* residing within the genome of each of the organism segregated on the basis of the non-pathogenic and pathogenic nature of the organism.

**Fig 8:**
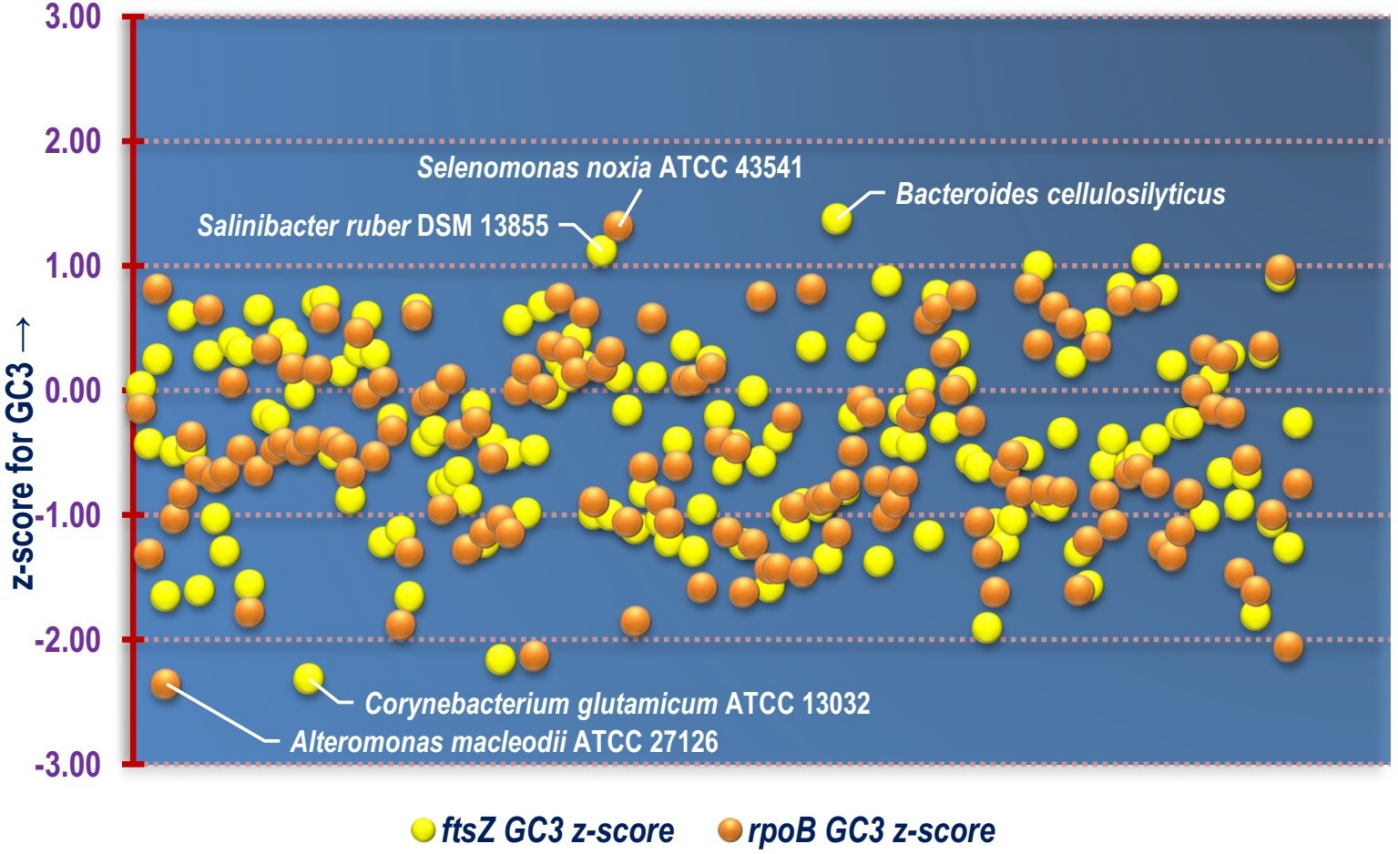
A scatter plot depicting the distribution of z-score for the GC3 of *ftsZ* and *rpoB* CDS of all the bacterial species included in this study. The name of the organisms with relatively higher and lower GC3 z-score have been labelled in the plot.

Out of the 143 organisms, the *ftsZ* CDS of organisms like *Fusobacterium nucleatum*, *Arcobacter butzleri* RM4018, *Staphylococcus aureus*, *Aerococcus viridans*, *Lactobacillus crispatus* ST1, *Enterococcus avium* ATCC 14025, *Lactococcus lactis* subsp. lactis Il1403, *Bacillus mycoides*, *Butyrivibrio proteoclasticus* B316 hve GC3 content which is substantially less (upto 66% lesser) than the mean genomic GC3 content. Barring *Lactococcus lactis* subsp. lactis Il1403, *Bacillus mycoides* and *Butyrivibrio proteoclasticus* B316, the remaining organisms are pathogenic in nature. This is an interesting observation which shows that the *ftsZ* CDS of these pathogenic bacteria are structured without significant bias towards G/C ending codons. Organisms like *Prochlorococcus marinus* str. AS9601, *Piscirickettsia salmonis* LF-89 and *Bacteroides cellulosilyticus* on the other hand, had significantly greater GC3 (20%, 29% and 40% respectively) in their *ftsZ* CDS compared to their genomic GC3 content. In terms of GC3, the percentage of genes within the genome of an organism with GC3 below that of *ftsZ* (S5 Table), depicted in Fig 7, was found to relatively differ in contrast to Nc. Similar trend was also visible in case of *rpoB*. About 10% of the total 143 organisms have more than 75% genes in their genome with GC3 greater than that of both *ftsZ* and *rpoB*. A graphical representation of the comparative z-score of *ftsZ* and *rpoB* GC3 is shown in Fig 8. The z-score data of GC3 is provided as supplementary information in S6 Table. Two Gram negative bacterial species *Bacteroides cellulosilyticus* and *Salinibacter ruber* DSM 13855 were found to demonstrate the maximal *ftsZ* GC3 z-score.

### Relative study of genomic GC content and *ftsZ* GC content

**Fig 9:**
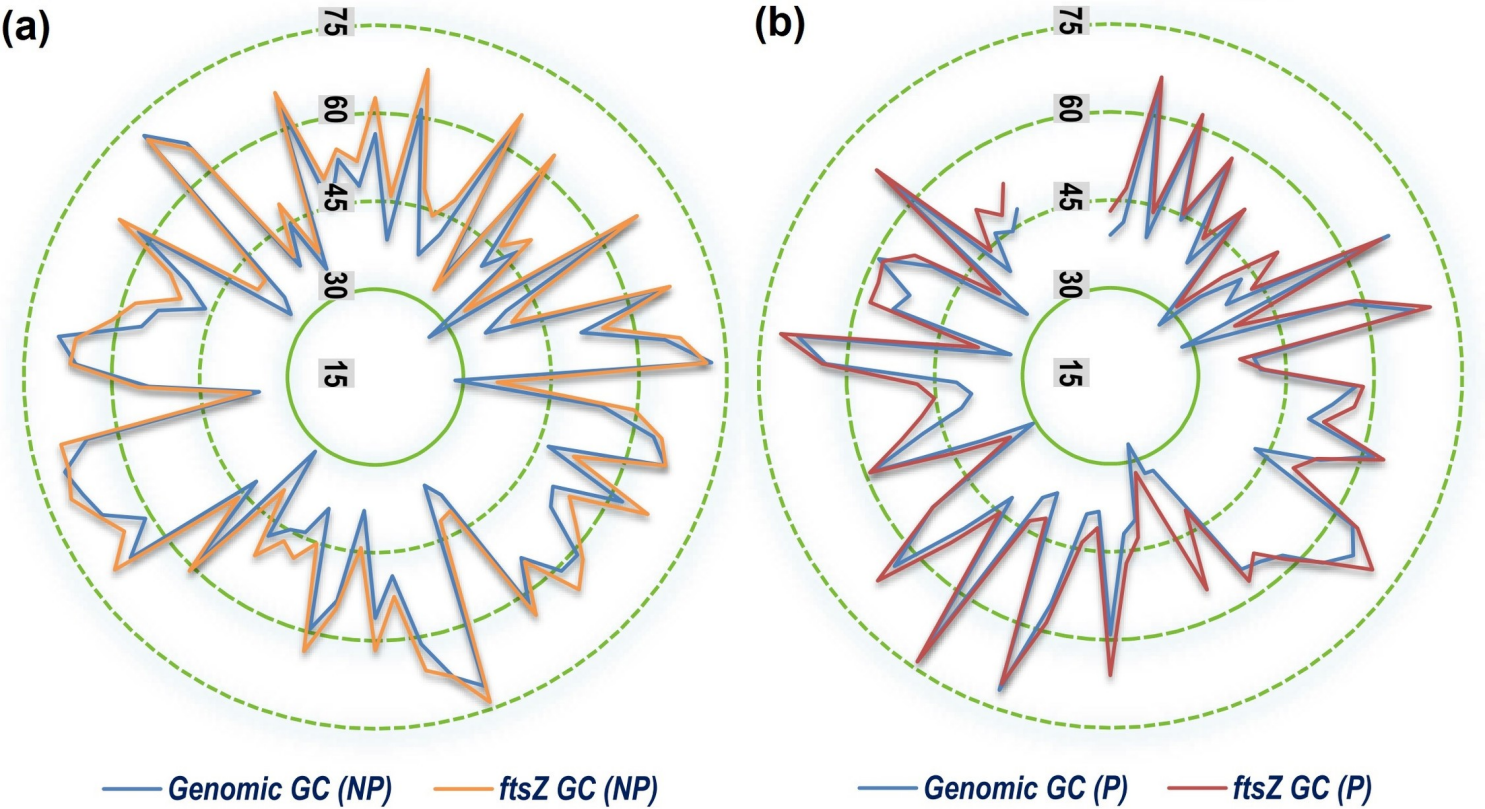
A radial plot comparing the genomic GC content with genic *ftsZ* GC content in (a) non-pathogenic [NP], and (b) pathogenic bacterial species.

The guanine-cytosine (GC) composition of bacterial genomes is a very important taxonomic marker from the genomics perspective. The GC content of a genome as well as that of a gene have been reported to be a significant genomic indicator for delineating covalently closed circular plasmid DNA from chromosomal DNA [99], and for distinguishing between vertically and horizontally transferred genes [100]. In our study we found that, out of the 143 organisms, the *ftsZ* CDS of 49 organisms depicted greater than 10% GC content variation in comparison to their genomic GC content. Among these organisms, *Coxiella burnetii* RSA 493, *Rickettsia conorii* str. Malish 7, *Staphylococcus aureus*, *Bacteroides cellulosilyticus*, *Fusobacterium nucleatum*, *Lactococcus lactis* subsp. lactis Il1403, *Anaerostipes hadrus* DSM 3319 and *Acinetobacter johnsonii* XBB1 demonstrates 15% greater usage of GC residues in their *ftsZ* CDS in comparison to the whole genome GC content. In comparison to the genomic GC content, a relatively greater usage of GC residues (more than 20% to 68%) was observed in the *ftsZ* CDS of *Francisella philomiragia* subsp. philomiragia ATCC 25017, *Staphylococcus capitis* subsp. capitis, *Clostridium butyricum*, *Prochlorococcus marinus* str. AS9601, *Buchnera aphidicola* str. APS, *Borrelia burgdorferi* B31 and *Flavobacterium hydatis*. The GC content of *ftsZ* CDS in comparison to the genomic GC of *Flavobacterium hydatis* was an extraordinarily 68% greater. On the other hand, the GC content of *ftsZ* CDS in comparison to the genomic GC content of organisms like *Bacillus mycoides*, *Capnocytophaga ochracea* DSM 7271, *Salinispora tropica* CNB-440, *Eikenella corrodens* ATCC 23834 and *Bordetella bronchiseptica* 253 was found to be 2% to 6% lower. A comparative graphical depiction of the genomic GC and genic *ftsZ* GC content segregated on the basis of lifestyle is given in Fig 9. A Mann-Whitney U test was conducted to statistically validate the difference between the genomic GC content and the *ftsZ* genic GC content of the 143 species. The results suggest that the genomic GC content and *ftsZ* GC content differs significantly (*U* = 8536.50, *p* = 0.016). All these findings evidently suggest that the nucleotide composition of the gene coding for FtsZ protein in a large number of species deviates significantly from their genomic GC content. The deviation of GC content of a coding sequence or a patch of nucleotides from the genomic GC content is a possible pointer towards horizontal gene transfer or HGT [101], and our analysis using Mann-Whitney U test also points in that direction.

### Relationship between GC1, GC2 and GC3

**Fig 10:**
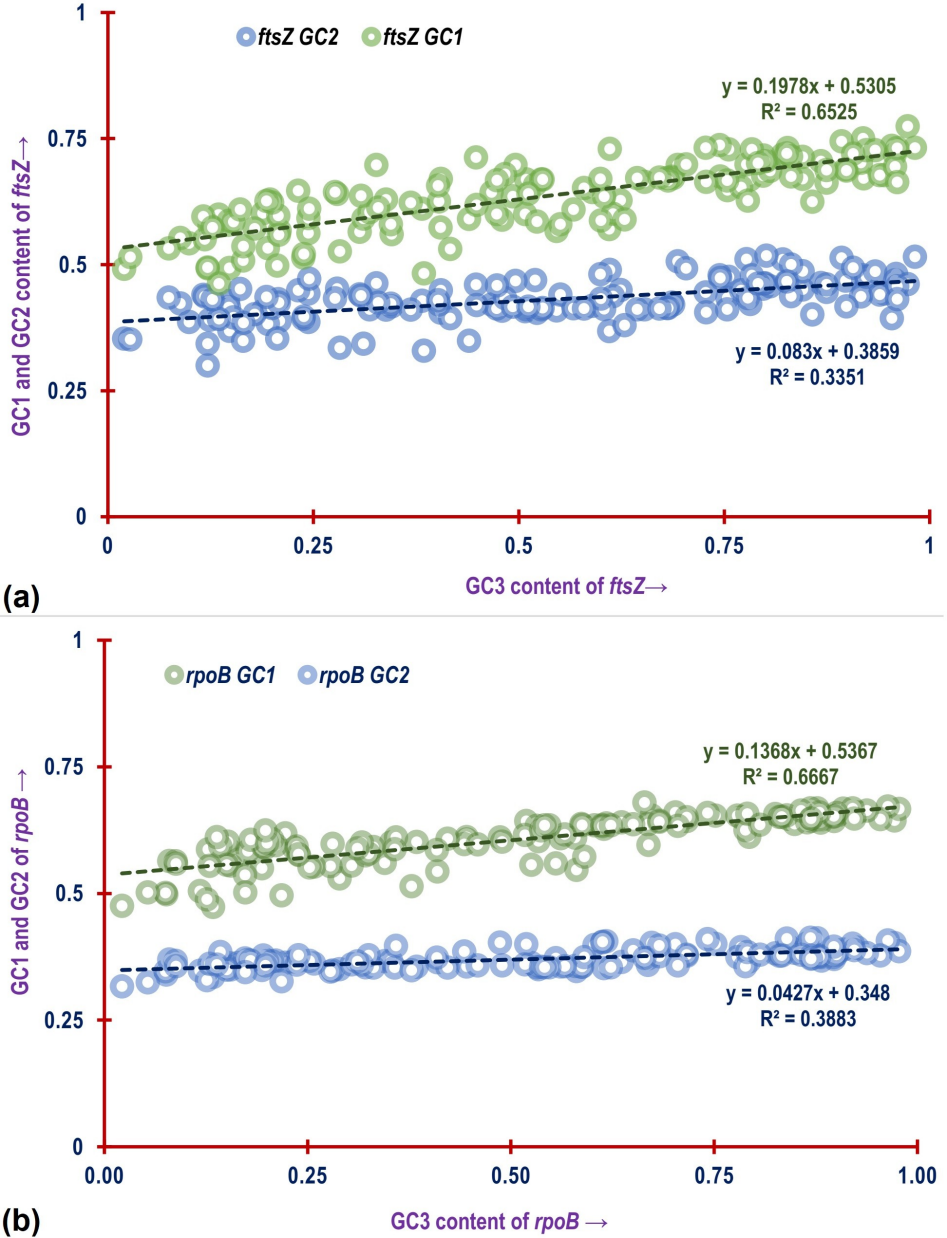
A plot showing the linear relationship of GC3 values with GC1 values and GC3 with GC2 values of (a) *ftsZ* and (b) *rpoB* CDS. The green and blue dashed line represents the trend line of the association between GC3-GC1 and GC3-GC2 respectively. The equation for the line and *R*^2^ value for GC3-GC1 and GC3-GC2 association is depicted in green and blue colours.

**Fig 11:**
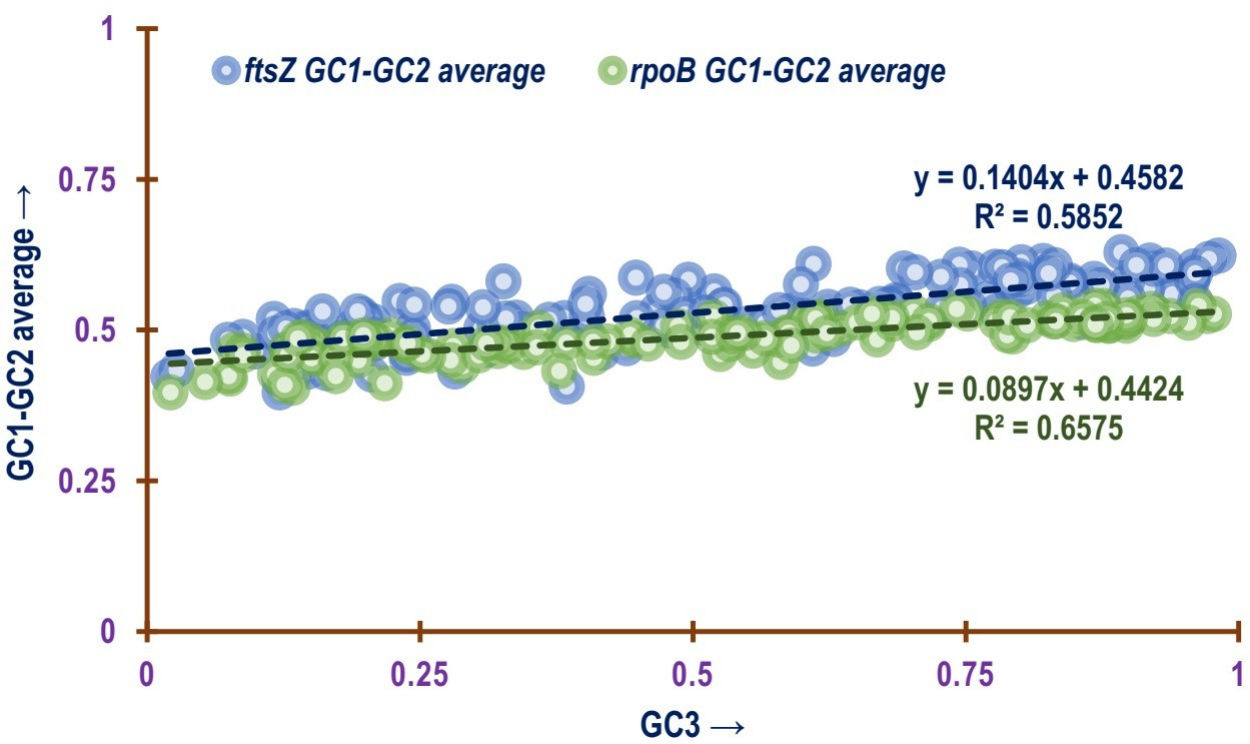
A plot showing the relationship of the average of GC1 and GC2 values (GC1-GC2) plotted against GC3 values of *ftsZ* and *rpoB* CDS of all the bacterial species involved in this study. The blue and green dashed line represents the trend of association between GC1-GC2 with GC3 of *ftsZ* and *rpoB* CDS respectively. The equation for the line and *R*^2^ value for *ftsZ* and *rpoB* CDS is depicted in blue and green colours respectively.

The relationship between GC1, GC2 and GC3 have been utilised in different studies to explore the mechanistic factor shaping coding usage pattern [78,102–104]. Utilizing Spearman’s Rank Order Correlation significant positive correlation was found to exist between the GC1 and GC2 of *ftsZ* (*ρ* = 0.754; p<0.01). Similar trend was also observed in the case of *rpoB* (*ρ* = 0.70; p<0.01). The linear relationship of GC3 with GC1 and GC3 with GC2 shown in Fig 10 for *ftsZ* CDS depicts a comparatively stronger positive association between GC3 and GC1 (*R*^2^ = 0.6525; p<0.01) with slope approaching 0. As seen in Fig 10, a similar trend is also reflected by the *rpoB* CDS. With respect to the relation between GC3 and GC2, both *ftsZ* (*R*^2^ = 0.335; p<0.01) and *rpoB* (*R*^2^ = 0.3883; p<0.01) demonstrated a comparatively weak association with a slope approaching relatively much closer to 0. The average of GC1 and GC2 was plotted against GC3 in a neutrality plot for the *ftsZ* CDS shown in Fig 11, and a significant positive correlation between the two is evident with a slope approaching 0 (*y* = 0.1404*x* + 0.4582; *R*^2^ = 0.5852, p<0.01) further suggesting that codon usage of *ftsZ* CDS is shaped by natural selection [104,105]. This trend was also found to be replicated by the *rpoB* CDS as depicted in Fig 11.

Analysis of the different codon usage parameters of the *ftsZ* CDS such as Nc, GC, GC1, GC2, GC3 and their interrelationships strongly suggest the fact that the codon usage pattern of *ftsZ* gene is relatively biased and is to a large extent shaped by forces of selection. Results obtained from the tandem analysis of a key housekeeping gene such as *rpoB* were found to be a mostly in line with that of *ftsZ*, emphasizing the significance of *ftsZ* as a key component in maintenance of bacterial cellular process such as cell division.

### Detection of ‘core’ set of codons used in structuring of *ftsZ* CDS

The individual usage frequency of the 61 sense codons from the 143 organisms were calculated (S4 Table). Out of the 61 sense codons, the two non-degenerate codons coding for methionine and tryptophan were eliminated. For the remaining 18 amino acids, the 59 codons were grouped in to their degenerate classes of 2, 3, 4 and 6 codons. This analysis was performed to find out if there exists a preferred set of ‘core’ codons for each of the amino acids used in structuring of the *ftsZ* CDS. A Kruskal-Wallis one way analysis of variance on ranks was carried out for the amino acids coded by 3, 4 and 6 codons, whereas Mann-Whitney Rank Sum test was used to test the codon preference in the two codon family amino acids. The results established the fact that, out of the 18 amino acids, the codons of three amino acids namely aspartic acid, histidine and alanine are randomly utilised on a global scale for structuring the *ftsZ* CDS. On the other hand, the codons for the remaining 15 amino acids show a non-random utilization pattern. These amino acids include cysteine, glutamine, phenylalanine, glycine, isoleucine, lysine, leucine, asparagine, proline, glutamine, arginine, serine, threonine, valine and tyrosine. Table 1 contains the Mann-Whitney U statistic and the H-value with degrees of freedom for the Kruskal-Wallis one way analysis of variance on ranks with their corresponding *p-*value obtained from the tests. Our analysis using both the above mentioned robust inferential statistical tools suggest that for all the 18 amino acids (except aspartic acid, histidine and alanine) the differences in the median values among the codon groups are greater than would be expected by chance and hence there is a statistically significant difference at *p =* <0.001 level. This is a substantial finding suggesting the antiquity and conservation of a preferred set of codons in structuring of a vital gene such as *ftsZ*.

**Table 1:** Mann-Whitney U statistic and the H-value with degrees of freedom for the Kruskal-Wallis one way ANOVA on ranks on codon usage to detect ‘core’ set of codons used in structuring of *ftsZ*.

*Sl*. *No*.	*Amino acid*	*Degenerate codon family*	*Mann-Whitney U statistic*	*H-value with degrees of freedom (df) for the Kruskal-Wallis one way analysis of variance on ranks*	*p*-*value*
*1*	**Cys**	2	8029.50	-	<0.001
*2*	**Glu**	2	5622.00	-	<0.001
*3*	**Phe**	2	8375.50	-	0.008
*4*	**Lys**	2	7552.00	-	<0.001
*5*	**Asn**	2	6426.00	-	<0.001
*6*	**Gln**	2	6384.00	-	<0.001
*7*	**His**	2	9211.00	-	0.145
*8*	**Asp**	2	9565.50	-	0.346
*9*	**Tyr**	2	8696.50	-	0.001
*10*	**Ile**	3	-	229.853, *df* = 2	<0.001
*11*	**Gly**	4	-	273.266, *df* = 3	<0.001
*12*	**Thr**	4	-	70.997, *df* = 3	<0.001
*13*	**Val**	4	-	49.583, *df* = 3	<0.001
*14*	**Ala**	4	-	3.777, *df* = 3	0.287
*15*	**Pro**	4	-	69.438, *df* = 3	<0.001
*16*	**Leu**	6	-	144.051, *df* = 5	<0.001
*17*	**Arg**	6	-	406.291, *df* = 5	<0.001
*18*	**Ser**	6	-	76.847, *df* = 5	<0.001

### Interrelation of *ftsZ* codon deployment with lifestyle and Gram nature of bacteria

Sixty one separate variance analysis tests called two factor (or two way) ANOVA was performed to find out how the two major factors namely lifestyle (pathogenic or non-pathogenic), Gram nature and interaction of these two factors influence the coding composition of the *ftsZ* CDS in the selected organisms at *p*<0.01 level of significance. A critical analysis of the results show that the compositional bias of eight codons coding for six amino acids is influenced mostly by the Gram nature of the organisms and in some instances by the interaction of lifestyle and Gram nature. In our study, we find that the compositional bias of the codons AUG (methionine), UCA (serine), UAU (tyrosine) and UAC (tyrosine) is influenced solely by the Gram nature of the organism. On the other hand, the compositional frequency of the codons GGA (glycine), CUU (leucine), CUG (leucine) and ACA (threonine) is influenced by the interaction between the Gram nature of the organism and their lifestyle preference of being either pathogenic or non-pathogenic. The two way ANOVA results suggest that the codon organization of the *ftsZ* CDS is determined largely by the Gram nature and pathogenic/non-pathogenic nature of the organisms, and it is a non-randomly constituted sequence in terms of codon deployment.

### Interrelation of amino acid deployment of *ftsZ* with lifestyle and Gram nature

To further comprehend the codon deployment pattern of the *ftsZ* CDS, a two way ANOVA was carried out by grouping the different codons according to the amino acids they code (for example alanine is coded by four codons and these four codons are clubbed into a single category to estimate the total frequency of alanine residues present in the CDS). Twenty discrete two way ANOVA analysis was carried out to find if the two factors namely lifestyle, Gram nature and interaction of these two factors influence the amino acid composition of the *ftsZ* CDS in the selected organisms (at *p*<0.01 level of significance) or, is the amino acid composition random in nature. All the post-hoc pairwise multiple comparison in the analysis was performed using the Holm-Sidak method of pairwise multiple comparison [106,107]. The results show that the compositional frequency of the amino acids glutamic acid, phenylalanine, leucine, valine, glutamine, threonine and tryptophan is influenced neither by the lifestyle nor the Gram nature of the organism. But, the frequency of the amino acids like aspartic acid, histidine, glycine, methionine, cysteine and tyrosine is influenced by the Gram nature of the organism (*p*<0.01 level). This shows that the compositional frequency of at least one amino acid from the four different chemical classes of amino acids is directly associated with the Gram nature of the bacteria. Another interesting observation is that the two sulphur containing amino acids methionine and cysteine are both involved in inducing compositional variability based on the wall nature of the bacterium. The hydroxymethyl side chain containing polar amino acid serine was found to be unique in the sense that a two factor ANOVA on composition frequency of serine detected that it is influenced both by the Gram nature and lifestyle of the organism. No amino acid other than serine was found to be influenced by the lifestyle of the organism. Thus, serine appears to be the only amino acid in the FtsZ protein which acts as a pointer to the lifestyle of the bacterial species considered in this study. In case of the compositional frequency of the remaining amino acids like alanine, isoleucine, proline, lysine, arginine and asparagine the effect of lifestyle was found to rest on the Gram nature of the organisms at *p*<0.01 level.

### Phylogenetic and cluster based analysis of *ftsZ* CDS

**Fig 12:**
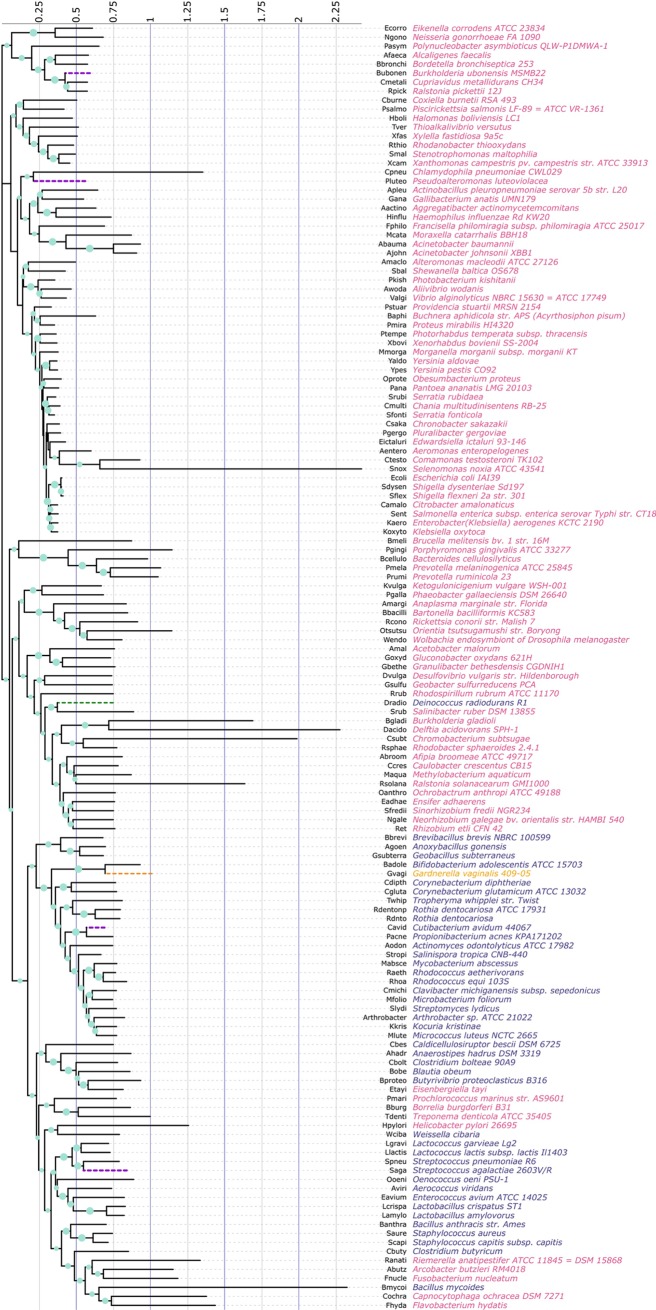
A phylogenetic tree depicting the evolutionary relationship between the 143 *ftsZ* CDS considered in this study constructed using MEGA X and annotated using iTOL. The evolutionary history was inferred using the Neighbor-Joining method and the optimal tree was obtained using 1000 bootstrap replicates. The tree is drawn to scale, with branch lengths representing evolutionary distances in the units of number of base substitutions per site. The evolutionary distances were computed using the Tajima-Nei method and are the rate variation among sites was modeled with a gamma distribution (shape parameter = 1). The name of the organisms have been given in colours to match with their Gram nature with blue representing *ftsZ* CDS of Gram positive whereas red represents *ftsZ* CDS of Gram negative bacterial species. The Gram variable bacterium has been depicted using orange. The green circles at the node represents the relative bootstrap support for each branch with large circle representing higher confidence level. The purple dashed line branches represents *ftsZ* CDS of those organisms that have been utilized for analysing the relationship between codons constituting *ftsZ* CDS and SSE of FtsZ proteins.

The level of identity existing within the 143 FtsZ protein coding genes determined using Clustal Omega [83] demonstrated tremendous variation, with identity ranging from 20% to 98% among the species considered in this study. This suggests a tremendous degree of heterogeneity existing within the *ftsZ* CDS across the different types of bacterial species residing within the eubacterial domain that have arisen in course of evolution over a long period of time. To infer the phylogenetic relationship of such a gene sequence that demonstrates substantial heterogeneity, a phylogenetic analysis was performed. A phylogenetic tree depicting the evolutionary relationship between the 143 *ftsZ* CDS given in Fig 12 show the *ftsZ* gene of *Gardnerella vaginalis* 409–05 to reside with the Gram positive bacterial species. This is quite understandable since many Gram positive bacteria under different conditions have been found to behave as Gram variable [108]. The *ftsZ* CDS of the Gram positive bacterium *Deinococcus radiodurans* R1 was found to peculiarly reside with the Gram negative bacteria. Another such obscure behaviour was displayed by the *ftsZ* of the Gram positive bacterium *Bacillus mycoides* which clustered with the Gram negative bacteria *Capnocytophaga ochracea* DSM 7271 and *Flavobacterium hydatis*. The *ftsZ* CDS of *Selenomonas noxia* ATCC 43541 was found to display the maximum number of substitutions followed by *Delftia acidovorans* SPH-1, *Bacillus mycoides*, *Chromobacterium subtsugae*, *Burkholderia gladioli*, *Ralstonia solanacearum* GMI1000 and *Chlamydophila pneumoniae* CWL029. Apart from *Bacillus mycoides*, all these species are Gram negative. The *ftsZ* CDS of many pathogenic Gram negative species were found to display least amount of evolutionary change measured in terms of substitutions. These include *Salmonella enterica* subsp. enterica serovar Typhi str. CT18, *Enterobacter*, *Shigella dysenteriae* Sd197, *Klebsiella oxytoca*, *Citrobacter amalonaticus*, *Yersinia pestis* CO92, etc.

The 143 *ftsZ* CDS were further subjected to clustering using CD-HIT with a 50% similarity threshold. A tabular account of the 17 clusters generated using CD-HIT along with the number of representative sequences for each cluster is given in Table 2. From the data given in Table 2, it is quite evident that the majority of the sequences are grouped together in the first two clusters which contains 43% of the total *ftsZ* CDS (41 sequences in Cluster 0 and 21 sequences in Cluster 1). The amino acid sequence of the corresponding *ftsZ* CDS representing the first four cluster i.e., *Pseudoalteromonas luteoviolacea* (Cluster 0), *Cutibacterium avidum* 44067 (Cluster 1), *Streptococcus agalactiae* 2603V/R (Cluster 2) and *Burkholderia ubonensis* MSMB22 (Cluster 3) were subjected to secondary structure prediction using SSpro 5.2 module of SCRATCH Protein Predictor (http://scratch.proteomics.ics.uci.edu/)[109]. SSpro catalogues three classes of secondary structures and based on that, the amino acid residues constituting the four FtsZ proteins have been identified as H (alpha helix), E (strand) and C (all the rest SSE). We have meticulously aligned the FtsZ amino acid sequences with the SSE mark-up sequence (S1 Fig) generated using Sspro 5.2. Using this alignment for each of the four representative sequence, we have identified the individual codons coding for the different amino acids within the three classes of SSE detected by Sspro (S2 Fig). We have analysed the RSCU values of the *ftsZ* CDS of *Pseudoalteromonas luteoviolacea*, *Cutibacterium avidum* 44067, *Streptococcus agalactiae* 2603V/R and *Burkholderia ubonensis* MSMB22 by splitting the sequences into the three different SSE classes (S7 Table).

**Table 2:** Clusters of *ftsZ* gene sequences generated using CD-HIT with a similarity threshold of 50 percent.

*Cluster at 50% identity*	No. of sequences in the cluster	Representative sequence	Length of the representative sequence
*Cluster 0*	41	*Pseudoalteromonas luteoviolacea*	418
*Cluster 1*	21	*Cutibacterium avidum* 44067	417
*Cluster 2*	9	*Streptococcus agalactiae* 2603V/R	419
*Cluster 3*	6	*Burkholderia ubonensis* MSMB22	399
*Cluster 4*	4	*Butyrivibrio proteoclasticus* B316	412
*Cluster 5*	3	*Acinetobacter johnsonii* XBB1	398
*Cluster 6*	2	*Neisseria gonorrhoeae* FA 1090	392
*Cluster 7*	2	*Geobacter sulfurreducens* PCA	383
*Cluster 8*	2	*Anaplasma marginale* str. Florida	412
*Cluster 9*	1	*Fusobacterium nucleatum*	360
*Cluster 10*	1	*Deinococcus radiodurans* R1	371
*Cluster 11*	1	*Helicobacter pylori* 26695	385
*Cluster 12*	1	*Arcobacter butzleri* RM4018	377
*Cluster 13*	1	*Chromobacterium subtsugae*	400
*Cluster 14*	1	*Ralstonia solanacearum* GMI1000	408
*Cluster 15*	1	*Borrelia burgdorferi* B31	399
*Cluster 16*	1	*Selenomonas noxia* ATCC 43541	412

### Comparative RSCU analysis of the helix, strand and other structural elements constituting residues of *ftsZ*

A RSCU analysis of the sense codons used for coding the amino acids of the FtsZ protein of *Pseudoalteromonas luteoviolacea*, *Cutibacterium avidum* 44067, *Streptococcus agalactiae* 2603V/R and *Burkholderia ubonensis* MSMB22, based on the type of SSE was performed. An amino acid wise description of the RSCU of the sixty one sense codons used in structuring of the *ftsZ* CDS is described below.

#### Non Polar amino acids

**Glycine**: In case of glycine, the residues constituting the helix in proteins are encoded by the codons GGU, GGA, GGG and GGC of which GGC is used by all the four species. In the strand region, *Cutibacterium* utilizes all the four codons whereas *Burkholderia* and *Pseudoalteromonas* use only two codons GGU and GGC. Likewise, *Streptococcus* also prefers the codons GGU and GGG only. This suggests that in these three bacterial species there is a preference towards a certain subset of codons in coding the glycine residues positioned in the strand regions. In all the remaining secondary structural elements, all the organisms are found to use GGU, GGG and GGC.

**Alanine**: The amino acid alanine is near universally encoded by GCU, GCC, GCA and GCG. In the helix region, GCG and GCC codons are used by all the four organisms. All the four codons are found to be employed by *Streptococcus*. But in the strand region, GCU and GCA are used only by *Streptococcus*. In the remaining regions, all the four codons are used randomly by all the organisms.

**Valine**: It is encoded by four codons GUU, GUC, GUA and GUG of which in the helix region, GUG is used by all the four organisms. In contrast to the helix region, in the strand region *Pseudoalteromonas* uses all the valine triplets whereas *Cutibacterium* and *Streptococcus* was found to use only three. In all the remaining regions, all the four organisms use the codons GUG and GUC.

**Methionine**: Since methionine is coded by a single codon AUG, we observed that for all the three regions, the codon AUG is preferred by all the four species.

**Leucine**: It is one of the three amino acids which is encoded by six different codons UUA, UUG, CUU, CUC, CUG and CUA. In the helix elements, the codon UUG was used by all the organisms whereas the codon CUA is totally absent in the helix region. In the strand region, *Burkholderia*, *Cutibacterium*, *Streptococcus* and *Pseudoalteromonas* was found to use a specific subset of leucine codons. No organism was found to use all the 6 codons. In the remaining regions, it was observed that the codon CUA is not used by any of the species. The codon CUG is used by three organisms except *Streptococcus*.

**Isoleucine**: In the helix regions, the codon AUC is used by all the organisms whereas AUA remains totally absent. But in the strand regions, codon AUA is used only by *Pseudoalteromonas* which also uses the other two codons AUU and AUC. *Burkholderia* uses only AUC but *Streptococcus* uses AUU and AUC. In the rest of the remaining regions, AUC is used by three of the organisms except *Streptococcus*. AUA is not used by any of the organisms.

**Proline:** In the helix regions, codon CCA is used by only two organisms–*Pseudoalteromonas* and *Streptococcus* whereas codon CCC is used by a single organism, *Cutibacterium*. Three organisms use the codon CCU except *Burkholderia*. In the strand regions, out of the four codons of proline, CCC is used by *Cutibacterium* and CCU by *Pseudoalteromonas*. The rest two codons aren’t used. In case of the remaining secondary structural elements, *Cutibacterium* is found to use all the four codons.

**Phenylalanine**: It is encoded by two codons–UUU and UUC. Considering the codon usage of the phenylalanine residues in the helix regions, the codon UUU is used by *Pseudoalteromonas* and *Streptococcus* whereas UUC is used by all the organisms except *Pseudoalteromonas*. But in strand elements, codon UUU is only used by *Streptococcus* and *Pseudoalteromonas*. The use of UUC is totally avoided here. In case of the remaining secondary structural elements, UUU codon is used by all the four species.

**Tryptophan**: The amino acid tryptophan is encoded by a single codon UGG in a near universal manner. In case of helix elements of *ftsZ* CDS, this amino acid is totally absent. In the strand elements, UGG is used only by *Streptococcus* whereas in the remaining elements, tryptophan is found to be used by *Burkholderia* and *Streptococcus*.

**Tyrosine**: In the helix regions, we found that the codon UAC is used by *Burkholderia* alone. Similarly *Pseudoalteromonas* use the codon UAU. In strand regions, UAU remains totally absent whereas UAC is used by *Burkholderia* alone. UAC is not used by *Pseudoalteromonas*. In the remaining regions, UAU is found to be used by the organisms *Streptococcus* and *Pseudoalteromonas*.

#### Polar basic amino acids

**Histidine**: In the helix regions, histidine is coded by CAC in three of the organisms except *Streptococcus*. Similarly, another codon CAU is preferred in the helix regions buy all the three organisms except *Cutibacterium*. In the extended strand regions, our analysis shows that the amino acid histidine is not used by any of the organisms. For the rest of the remaining secondary structural elements CAU is preferred by all the four organisms except *Streptococcus* which uses CAC.

**Lysine**: This amino acid is encoded by two codons—AAA and AAG. In the helix regions lysine is found to be coded by the homo triplet AAA in the studied organisms except *Burkholderia*. AAG was found to be employed by all the four organisms. In the strand regions, the triplet AAA is used by the organisms *Pseudoalteromonas* and *Streptococcus* whereas AAG is preferred by *Burkholderia* and *Cutibacterium*. For the remaining secondary structural elements, the preference for AAA is restricted to *Pseudoalteromonas* and *Streptococcus*, a scenario exactly similar to the strand region.

**Arginine**: This is one of the three amino acid which is encoded by six degenerate codons–CGU, CGC, CGA, CGG, AGA and AGG. In the helix regions, none of the four organisms were found to use the codon CGA. The remaining organisms display preference towards the use of specific subset of codons. In the strand regions only three codons AGA, CGC and CGU are used out of the six. This suggests the preference of the organism towards specific codons for encoding the amino acids that have the propensity to be included in the strand regions of FtsZ protein. In the remaining structural elements, out of the six codons two are totally absent and this are CGG and AGA. The codon CGU is found to be used by *Cutibacterium*, *Pseudoalteromonas*, *Streptococcus* and comparatively in lesser frequency by *Burkholderia*.

#### Polar acidic amino acids

**Aspartic acid**: In the helix region, all the four organisms use the codons GAU and GAC, but the frequency of GAU usage by *Cutibacterium* is very low. In contrast to the helix regions, in strand regions we found that *Pseudoalteromonas* does not use aspartic acid. *Streptococcus* use GAU alone whereas GAC is used by *Burkholderia* and *Cutibacterium*.

**Glutamic acid**: This amino acid is represented by the codons GAA and GAG. In the helix, GAG is used by all the four organisms whereas GAA is used by all except *Cutibacterium*. In the strand regions, GAA is used by *Burkholderia* and *Streptococcus* whereas GAG is used by *Cutibacterium* alone. Both the codons are found to remain absent in *Pseudoalteromonas*. In the rest of the structural elements, GAG is preferred by all the organisms.

#### Polar neutral amino acids

**Serine**: It is encoded by six codons—UCU, UCC, UCA, UCG, AGU, AGC. A preferential usage of certain codons encoding the different amino residues constituting the different structural elements was also observed in case of this amino acid. In the helix regions, AGC is used by all the organisms except *Cutibacterium*. The codon AGU UCA and UCC is found to be preferred by *Streptococcus* and *Cutibacterium*. *Burkholderia* and *Cutibacterium* was found to prefer UCG, whereas *Pseudoalteromonas* and *Streptococcus* use UCU. The use of the codon UCU by *Pseudoalteromonas* was found to be comparatively higher than the rest of the organisms. In the strand regions, the codon AGU and UCU were found to be avoided by all the four organisms. In the rest of the structural elements, AGC was found to be preferred by all the four organisms. All the four organisms use the codon UCG, but in *Burkholderia* the frequency of usage is relatively greater.

**Threonine**: In the helix elements, ACC and ACA are preferred by three organisms. ACC remains absent in *Pseudoalteromonas* whereas ACA is absent in *Burkholderia*. All the four organisms preferentially use the codon ACG. In the extended strand elements, ACG is used by all the organisms whereas ACA and ACU codons are used by *Streptococcus* and *Pseudoalteromonas* and ACC is preferred by *Burkholderia* and *Cutibacterium*.

**Asparagine**: This amino acid is encoded in general by two codons–AAU and AAC. In the helix regions, AAC is preferred by all the organisms. Likewise codon AAU is also used by all the organisms. But in the strand region, AAU is used by two organisms *Pseudoalteromonas* and *Streptococcus*. The codon AAC is employed by all the organisms except *Pseudoalteromonas*. In the remaining structural elements, we did not observe any fixed preference for a particular codon in the organisms considered in this analysis.

**Glutamine**: In case of helix regions, codons CAA and CAG are used by three organisms. The use of the amino acid glutamine in the helix region was absent in *Cutibacterium*. CAG is used by *Burkholderia*, *Pseudoalteromonas and Streptococcus*. The codon CAA was not used by any of the organisms in the strand regions. In the other secondary structural elements, the codon CAG is used by all the organisms whereas the codon CAA is used by all the organisms except *Burkholderia*.

**Cysteine**: In the helix elements, the codon UGU was avoided by all the organisms. The use of this sulphur containing amino acid in the helix regions of *Burkholderia* and *Cutibacterium* are found to be fulfilled by the codon UGC. Apart from the helix structural elements, cysteine was found to be totally absent in the other secondary structural elements in all the four organisms.

Our study clearly shows that a differential RSCU pattern is evident in the coding nature of the various SSE of the FtsZ proteins from different bacteria. It may be suggested that this variation could be attributed to the differential folding pattern of the different domain region of the FtsZ protein. The FtsZ protein has two major domains—one is the GTPase domain and the other is the C-terminal domain. Our findings suggest that the use of specific codons coding for the amino acids in the different SSE of the FtsZ protein is less organism specific but more codon specific. The helix regions demonstrates a comparative higher bias towards use of specific codons in coding the amino acids than the strand or the other SSE regions.

## Conclusions

The FtsZ protein is ubiquitous in bacteria and plays a vital role in bacterial cell division. From the evolutionary stand point it might be regarded as the counterpart of the eukaryotic tubulin protein. Our study of the gene sequences coding for FtsZ from 142 bacterial species demonstrated that about one third of the selected organisms depicted more than ten percent GC variation in their *ftsZ* CDS compared to their genomic GC content. Thus, our study clearly suggest that the nucleotide composition of the gene coding for FtsZ protein in a large number of species deviates significantly from their genomic GC content. The codon usage pattern demonstrated that the *ftsZ* gene of about 93% of the organism show relatively biased codon usage profile and the same is largely influenced by natural selection. Parallel analysis of the housekeeping gene *rpoB* also demonstrated the resemblance of *ftsZ* codon usage pattern with a housekeeping gene. In this study, we have also captured the existence of a ‘core’ set of codons in the structuring of the *ftsZ* gene despite the presence of a varying degree of identity among the *ftsZ* sequences. This is probably due to the constraint exerted by nature to maintain form and function in an important physiological protein like FtsZ that plays a major role in a critical bacterial cellular process like cell division. By the utilization of inferential statistical methods such as a two way ANOVA, we were able to capture the influence of Gram nature of the bacteria and their lifestyle pattern on the amino acid compositional frequency of the FtsZ protein. Finally, a phylogenetic and cluster based analysis followed by an amino acid wise comparative RSCU analysis of the different secondary structural elements of the FtsZ protein tied with the *ftsZ* CDS, demonstrated the presence of bias towards specific triplet codons coding the amino acids of the different secondary structural elements of a multi domain protein like FtsZ. In conclusion, it may be stated that the *ftsZ* gene coding for an indispensable cell division protein called FtsZ in a large number of bacteria, differing in terms of cellular morphology, physiology, biochemistry and a host of other features displays a significantly biased codon usage pattern with an extremely inflated GC content. Along with the existence of a preferred ‘core’ set of codons, the different SSE of the multi-domain FtsZ protein was also found to display bias towards specific synonymous codons particularly in the helix and strand regions. All these suggest that in an indispensable and vital protein such as FtsZ, there is an inherent tendency to maintain form for optimized performance in spite of the extrinsic variability in coding features.

## Supporting information

S1 Table Details regarding NCBI genome accession number, links pertaining to the genome information page, reference to the information regarding the organism in the corresponding volumes of Bergey’s Manual of Systematic Bacteriology and locus tag/protein id of *ftsZ* and *rpoB* gene sequences used in this study. (TXT)

S2 Table Details of the different bacterial species along with their lifestyle, Gram nature and codon usage attributes of the *ftsZ* gene considered in the study. (TXT)

S3 Table Details of the different bacterial species along with codon usage attributes of the *rpoB* gene considered in the study. (DOC)

S4 Table Codon count data for the 143 *ftsZ* CDS used in statistical analysis. (TXT)

S5 Table Percentage of genes with Nc and GC3 less than that of *ftsZ* and *rpoB* residing within the genome of the 143 bacterial species considered in this study. (XLS)

S6 Table Data showing z-score of Nc and GC3 of *ftsZ* and *rpoB* CDS. (XLS)

S7 Table RSCU values of the *ftsZ* CDS of *Pseudoalteromonas luteoviolacea*, *Cutibacterium avidum* 44067, *Streptococcus agalactiae* 2603V/R and *Burkholderia ubonensis* MSMB22 generated by splitting the sequences into three different secondary structural element classes. (XLS)

S1 MSA Multiple sequence alignment data of the 143 *ftsZ* sequences in FASTA format generated using Clustal Omega. (TXT)

S1 Phylogeny Phylogenetic tree of *ftsZ* gene along with bootstrap support in Newick format. (TXT)

S1 Fig The amino acid (Aa) sequences of FtsZ protein from the four bacterial species is shown here in conjunction with the type of secondary structural elements (SSE) each of the amino acids are predicted to constitute. (JPG)

S2 Fig The CDS of *ftsZ* from the four bacterial species is depicted here. (JPG)
