# Supplementary material for: Comparative in silico analysis of ftsZ gene from different bacteria reveals the preference for core set of codons in coding sequence structuring and secondary structural elements determination
Source: PLoS One. 2019 Dec 16;14(12):e0219231. doi: 10.1371/journal.pone.0219231 (PMC6913975; doi:10.1371/journal.pone.0219231)
Supplement: S3 Table — (DOC) [file pone.0219231.s003.doc]

**S3_Table:** Details of the different codon usage attributes of *rpoB* gene considered in the study.

| ***Organism*** | ***rpoB* Nc** | ***rpoB* GC** | ***rpoB* GC1** | ***rpoB* GC2** | ***rpoB* GC3** |
| --- | --- | --- | --- | --- | --- |
| *Acetobacter malorum* | 38.49 | 0.3849 | 0.65349 | 0.40259 | 0.67 |
| *Acinetobacter baumannii* | 36.86 | 0.3686 | 0.58547 | 0.36244 | 0.15 |
| *Acinetobacter johnsonii XBB1* | 38.67 | 0.3867 | 0.59061 | 0.3661 | 0.23 |
| *Actinobacillus pleuropneumoniae serovar 5b str. L20* | 36.53 | 0.3653 | 0.56069 | 0.36634 | 0.34 |
| *Actinomyces odontolyticus ATCC 17982* | 28.57 | 0.2857 | 0.66782 | 0.41156 | 0.88 |
| *Aerococcus viridans* | 36.12 | 0.3612 | 0.567 | 0.36265 | 0.16 |
| *Aeromonas enteropelogenes* | 33.53 | 0.3353 | 0.63663 | 0.3589 | 0.67 |
| *Afipia broomeae ATCC 49717* | 30.82 | 0.3082 | 0.64385 | 0.37218 | 0.87 |
| *Aggregatibacter actinomycetemcomitans* | 38.26 | 0.3826 | 0.55696 | 0.36262 | 0.53 |
| *Alcaligenes faecalis* | 38.33 | 0.3833 | 0.64041 | 0.3647 | 0.64 |
| *Aliivibrio wodanis* | 38.14 | 0.3814 | 0.60238 | 0.35517 | 0.15 |
| *Alteromonas macleodii ATCC 27126* | 38.54 | 0.3854 | 0.61876 | 0.36337 | 0.22 |
| *Anaerostipes hadrus DSM 3319* | 38.61 | 0.3861 | 0.55529 | 0.34992 | 0.16 |
| *Anaplasma marginale str. Florida* | 54.38 | 0.5438 | 0.5465 | 0.34679 | 0.58 |
| *Anoxybacillus gonensis* | 46.66 | 0.4666 | 0.60067 | 0.36731 | 0.35 |
| *Arcobacter butzleri RM4018* | 30.55 | 0.3055 | 0.47578 | 0.3167 | 0.02 |
| *Arthrobacter sp. ATCC 21022* | 30.82 | 0.3082 | 0.65355 | 0.40034 | 0.75 |
| *Bacillus anthracis str. Ames* | 41.39 | 0.4139 | 0.58404 | 0.36842 | 0.18 |
| *Bacillus mycoides* | 42.45 | 0.4245 | 0.58998 | 0.36927 | 0.20 |
| *Bacteroides cellulosilyticus* | 40.36 | 0.4036 | 0.5295 | 0.3509 | 0.29 |
| *Bartonella bacilliformis KC583* | 40.03 | 0.4003 | 0.59538 | 0.36777 | 0.21 |
| *Bifidobacterium adolescentis ATCC 15703* | 27.48 | 0.2748 | 0.64751 | 0.39623 | 0.84 |
| *Blautia obeum* | 42.45 | 0.4245 | 0.59829 | 0.3481 | 0.28 |
| *Bordetella bronchiseptica 253* | 30.87 | 0.3087 | 0.65482 | 0.38506 | 0.90 |
| *Brevibacillus brevis NBRC 100599* | 46.14 | 0.4614 | 0.60678 | 0.36356 | 0.45 |
| *Brucella melitensis bv. 1 str. 16M* | 36.18 | 0.3618 | 0.64296 | 0.37736 | 0.72 |
| *Buchnera aphidicola str. APS (Acyrthosiphon pisum)* | 37.95 | 0.3795 | 0.47357 | 0.33507 | 0.13 |
| *Burkholderia gladioli* | 28.6 | 0.286 | 0.64307 | 0.3708 | 0.90 |
| *Burkholderia ubonensis MSMB22* | 28.86 | 0.2886 | 0.64646 | 0.36742 | 0.88 |
| *Butyrivibrio proteoclasticus B316* | 37.71 | 0.3771 | 0.58714 | 0.34857 | 0.13 |
| *Caldicellulosiruptor bescii DSM 6725* | 44.52 | 0.4452 | 0.55004 | 0.34906 | 0.19 |
| *Capnocytophaga ochracea DSM 7271* | 43.19 | 0.4319 | 0.55433 | 0.34567 | 0.28 |
| *Caulobacter crescentus CB15* | 26.85 | 0.2685 | 0.66765 | 0.38909 | 0.91 |
| *Chania multitudinisentens RB-25* | 41.14 | 0.4114 | 0.60908 | 0.35592 | 0.56 |
| *Chlamydophila pneumoniae CWL029* | 46.43 | 0.4643 | 0.55307 | 0.36153 | 0.26 |
| *Chromobacterium subtsugae* | 28.04 | 0.2804 | 0.64009 | 0.37428 | 0.88 |
| *Chronobacter sakazakii* | 33.67 | 0.3367 | 0.63849 | 0.40554 | 0.62 |
| *Citrobacter amalonaticus* | 36.2 | 0.362 | 0.63738 | 0.35517 | 0.59 |
| *Clostridium bolteae 90A9* | 43.54 | 0.4354 | 0.61004 | 0.35676 | 0.58 |
| *Clostridium butyricum* | 34.6 | 0.346 | 0.49838 | 0.33981 | 0.08 |
| *Comamonas testosteroni TK102* | 30.5 | 0.305 | 0.64916 | 0.37929 | 0.79 |
| *Corynebacterium diphtheriae* | 36.5 | 0.365 | 0.63466 | 0.39847 | 0.60 |
| *Corynebacterium glutamicum ATCC 13032* | 36.54 | 0.3654 | 0.64322 | 0.39966 | 0.52 |
| *Coxiella burnetii RSA 493* | 51.53 | 0.5153 | 0.55951 | 0.37228 | 0.56 |
| *Cupriavidus metallidurans CH34* | 29.73 | 0.2973 | 0.65668 | 0.38203 | 0.83 |
| *Cutibacterium avidum 44067* | 30.6 | 0.306 | 0.65 | 0.39397 | 0.78 |
| *Deinococcus radiodurans R1* | 29.98 | 0.2998 | 0.66017 | 0.39322 | 0.93 |
| *Delftia acidovorans SPH-1* | 29.83 | 0.2983 | 0.64697 | 0.3771 | 0.88 |
| *Desulfovibrio vulgaris str. Hildenborough* | 31.95 | 0.3195 | 0.63729 | 0.3831 | 0.85 |
| *Eikenella corrodens ATCC 23834* | 42.17 | 0.4217 | 0.59613 | 0.37231 | 0.67 |
| *Eisenbergiella tayi* | 44.74 | 0.4474 | 0.60317 | 0.35079 | 0.53 |
| *Ensifer adhaerens* | 30.26 | 0.3026 | 0.65362 | 0.37464 | 0.78 |
| *Enterobacter(Klebsiella) aerogenes KCTC 2190* | 34.88 | 0.3488 | 0.63515 | 0.35517 | 0.59 |
| *Enterococcus avium ATCC 14025* | 40.58 | 0.4058 | 0.57593 | 0.36763 | 0.24 |
| *Escherichia coli IAI39* | 35.92 | 0.3592 | 0.63366 | 0.35443 | 0.53 |
| *Flavobacterium hydatis* | 36.48 | 0.3648 | 0.50118 | 0.34618 | 0.08 |
| *Francisella philomiragia subsp. philomiragia ATCC 25017* | 37.78 | 0.3778 | 0.50478 | 0.34731 | 0.12 |
| *Fusobacterium nucleatum* | 32.29 | 0.3229 | 0.50211 | 0.32489 | 0.05 |
| *Gallibacterium anatis UMN179* | 40.59 | 0.4059 | 0.55498 | 0.36033 | 0.30 |
| *Gardnerella vaginalis 409-05* | 39.69 | 0.3969 | 0.6106 | 0.39697 | 0.36 |
| *Geobacillus subterraneus* | 39.46 | 0.3946 | 0.61209 | 0.36608 | 0.79 |
| *Geobacter sulfurreducens PCA* | 38.63 | 0.3863 | 0.62509 | 0.35667 | 0.79 |
| *Gluconobacter oxydans 621H* | 36.86 | 0.3686 | 0.66022 | 0.39986 | 0.70 |
| *Granulibacter bethesdensis CGDNIH1* | 36.79 | 0.3679 | 0.65876 | 0.4102 | 0.74 |
| *Haemophilus influenzae Rd KW20* | 37.34 | 0.3734 | 0.55134 | 0.36682 | 0.25 |
| *Halomonas boliviensis LC1* | 41.1 | 0.411 | 0.63701 | 0.3879 | 0.65 |
| *Ketogulonicigenium vulgare WSH-001* | 37.12 | 0.3712 | 0.65763 | 0.38445 | 0.79 |
| *Klebsiella oxytoca* | 35.53 | 0.3553 | 0.6277 | 0.35294 | 0.61 |
| *Kocuria kristinae* | 25.39 | 0.2539 | 0.6442 | 0.40188 | 0.97 |
| *Lactobacillus amylovorus* | 31.06 | 0.3106 | 0.56425 | 0.36985 | 0.08 |
| *Lactobacillus crispatus ST1* | 33.14 | 0.3314 | 0.56389 | 0.36686 | 0.09 |
| *Lactococcus garvieae Lg2* | 37.15 | 0.3715 | 0.59231 | 0.37761 | 0.20 |
| *Lactococcus lactis subsp. lactis Il1403* | 36.6 | 0.366 | 0.59315 | 0.38429 | 0.14 |
| *Methylobacterium aquaticum* | 27.07 | 0.2707 | 0.66691 | 0.38618 | 0.98 |
| *Microbacterium foliorum* | 29.28 | 0.2928 | 0.64041 | 0.40154 | 0.86 |
| *Micrococcus luteus NCTC 2665* | 26 | 0.26 | 0.67151 | 0.40719 | 0.96 |
| *Moraxella catarrhalis BBH18* | 41.3 | 0.413 | 0.57551 | 0.35411 | 0.32 |
| *Morganella morganii subsp. morganii KT* | 37.2 | 0.372 | 0.61579 | 0.36039 | 0.52 |
| *Mycobacterium abscessus* | 31.12 | 0.3112 | 0.65444 | 0.41126 | 0.84 |
| *Neisseria gonorrhoeae FA 1090* | 41.69 | 0.4169 | 0.57286 | 0.37473 | 0.59 |
| *Neorhizobium galegae bv. orientalis str. HAMBI 540* | 32.61 | 0.3261 | 0.63597 | 0.37926 | 0.81 |
| *Obesumbacterium proteus* | 40.27 | 0.4027 | 0.61132 | 0.35592 | 0.45 |
| *Ochrobactrum anthropi ATCC 49188* | 36.07 | 0.3607 | 0.64369 | 0.37591 | 0.68 |
| *Oenococcus oeni PSU-1* | 43.34 | 0.4334 | 0.59182 | 0.35392 | 0.33 |
| *Orientia tsutsugamushi str. Boryong* | 36.54 | 0.3654 | 0.48839 | 0.32874 | 0.13 |
| *Pantoea ananatis LMG 20103* | 39.43 | 0.3943 | 0.62472 | 0.35517 | 0.54 |
| *Phaeobacter gallaeciensis DSM 26640* | 37.46 | 0.3746 | 0.65047 | 0.37926 | 0.72 |
| *Photobacterium kishitanii* | 38.44 | 0.3844 | 0.6073 | 0.36587 | 0.21 |
| *Photorhabdus temperata subsp. thracensis* | 41.19 | 0.4119 | 0.60164 | 0.35517 | 0.38 |
| *Piscirickettsia salmonis LF-89 = ATCC VR-1361* | 48.03 | 0.4803 | 0.5975 | 0.38558 | 0.44 |
| *Pluralibacter gergoviae* | 33.51 | 0.3351 | 0.63366 | 0.35815 | 0.71 |
| *Polynucleobacter asymbioticus QLW-P1DMWA-1* | 43.71 | 0.4371 | 0.57864 | 0.3782 | 0.33 |
| *Porphyromonas gingivalis ATCC 33277* | 58.04 | 0.5804 | 0.54331 | 0.36457 | 0.41 |
| *Prevotella melaninogenica ATCC 25845* | 36.76 | 0.3676 | 0.55669 | 0.34961 | 0.15 |
| *Prevotella ruminicola 23* | 36.09 | 0.3609 | 0.58773 | 0.35405 | 0.36 |
| *Prochlorococcus marinus str. AS9601* | 41.31 | 0.4131 | 0.53643 | 0.37158 | 0.17 |
| *Propionibacterium acnes KPA171202* | 38.35 | 0.3835 | 0.64138 | 0.39741 | 0.68 |
| *Proteus mirabilis HI4320* | 39.39 | 0.3939 | 0.5927 | 0.35964 | 0.24 |
| *Providencia stuartii MRSN 2154* | 39.3 | 0.393 | 0.60089 | 0.35964 | 0.32 |
| *Pseudoalteromonas luteoviolacea* | 38.56 | 0.3856 | 0.60581 | 0.36736 | 0.18 |
| *Ralstonia pickettii 12J* | 31.46 | 0.3146 | 0.65084 | 0.37692 | 0.83 |
| *Ralstonia solanacearum GMI1000* | 29.57 | 0.2957 | 0.65157 | 0.37473 | 0.90 |
| *Rhizobium etli CFN 42* | 33.13 | 0.3313 | 0.65145 | 0.37754 | 0.83 |
| *Rhodanobacter thiooxydans* | 27.01 | 0.2701 | 0.64672 | 0.37779 | 0.95 |
| *Rhodobacter sphaeroides 2.4.1* | 29.6 | 0.296 | 0.66401 | 0.38244 | 0.94 |
| *Rhodococcus aetherivorans* | 28.18 | 0.2818 | 0.66809 | 0.4012 | 0.92 |
| *Rhodospirillum rubrum ATCC 11170* | 31.4 | 0.314 | 0.64778 | 0.38809 | 0.92 |
| *Rickettsia conorii str. Malish 7* | 42.12 | 0.4212 | 0.49636 | 0.32678 | 0.22 |
| *Riemerella anatipestifer ATCC 11845 = DSM 15868* | 40.93 | 0.4093 | 0.50235 | 0.34483 | 0.17 |
| *Rothia dentocariosa* | 36.58 | 0.3658 | 0.63504 | 0.40342 | 0.61 |
| *Rothia dentocariosa ATCC 17931* | 37.93 | 0.3793 | 0.63286 | 0.40486 | 0.61 |
| *Salinispora tropica CNB-440* | 31.82 | 0.3182 | 0.66783 | 0.41259 | 0.87 |
| *Salmonella enterica subsp. enterica serovar Typhi str. CT18* | 36.53 | 0.3653 | 0.6344 | 0.35443 | 0.62 |
| *Selenomonas noxia ATCC 43541* | 34.77 | 0.3477 | 0.66399 | 0.37931 | 0.86 |
| *Serratia fonticola* | 38.29 | 0.3829 | 0.61653 | 0.35666 | 0.57 |
| *Serratia rubidaea* | 35.24 | 0.3524 | 0.67964 | 0.37275 | 0.66 |
| *Shewanella baltica OS678* | 41.56 | 0.4156 | 0.58929 | 0.36607 | 0.31 |
| *Shigella dysenteriae Sd197* | 35.89 | 0.3589 | 0.6344 | 0.35443 | 0.55 |
| *Shigella flexneri 2a str. 301* | 35.97 | 0.3597 | 0.6344 | 0.35443 | 0.54 |
| *Sinorhizobium fredii NGR234* | 30.94 | 0.3094 | 0.64808 | 0.37726 | 0.87 |
| *Staphylococcus aureus* | 35.04 | 0.3504 | 0.55912 | 0.35642 | 0.09 |
| *Staphylococcus capitis subsp. capitis* | 36.95 | 0.3695 | 0.5549 | 0.36149 | 0.13 |
| *Stenotrophomonas maltophilia* | 25.75 | 0.2575 | 0.65921 | 0.37906 | 0.90 |
| *Streptococcus agalactiae 2603V/R* | 35.53 | 0.3553 | 0.61158 | 0.3599 | 0.14 |
| *Streptococcus pneumoniae R6* | 38.36 | 0.3836 | 0.59984 | 0.36647 | 0.20 |
| *Streptomyces lydicus* | 28.71 | 0.2871 | 0.65289 | 0.39535 | 0.90 |
| *Thioalkalivibrio versutus* | 31.86 | 0.3186 | 0.66029 | 0.37574 | 0.88 |
| *Treponema denticola ATCC 35405* | 54.21 | 0.5421 | 0.51455 | 0.34846 | 0.38 |
| *Tropheryma whipplei str. Twist* | 54.99 | 0.5499 | 0.6106 | 0.40286 | 0.49 |
| *Vibrio alginolyticus NBRC 15630 = ATCC 17749* | 35.88 | 0.3588 | 0.62472 | 0.35815 | 0.20 |
| *Weissella cibaria* | 31.19 | 0.3119 | 0.57759 | 0.37842 | 0.24 |
| *Xanthomonas campestris pv. campestris str. ATCC 33913* | 29.12 | 0.2912 | 0.64769 | 0.37104 | 0.87 |
| *Xenorhabdus bovienii SS-2004* | 40.94 | 0.4094 | 0.60834 | 0.35369 | 0.42 |
| *Xylella fastidiosa 9a5c* | 46.1 | 0.461 | 0.58603 | 0.37365 | 0.41 |
| *Yersinia aldovae* | 42.47 | 0.4247 | 0.60238 | 0.35815 | 0.49 |
| *Yersinia pestis CO92* | 42.47 | 0.4247 | 0.59717 | 0.35815 | 0.46 |
