# Supplementary figures and images for: Comparative in silico analysis of ftsZ gene from different bacteria reveals the preference for core set of codons in coding sequence structuring and secondary structural elements determination
Source: PLoS One. 2019 Dec 16;14(12):e0219231. doi: 10.1371/journal.pone.0219231 (PMC6913975; doi:10.1371/journal.pone.0219231)

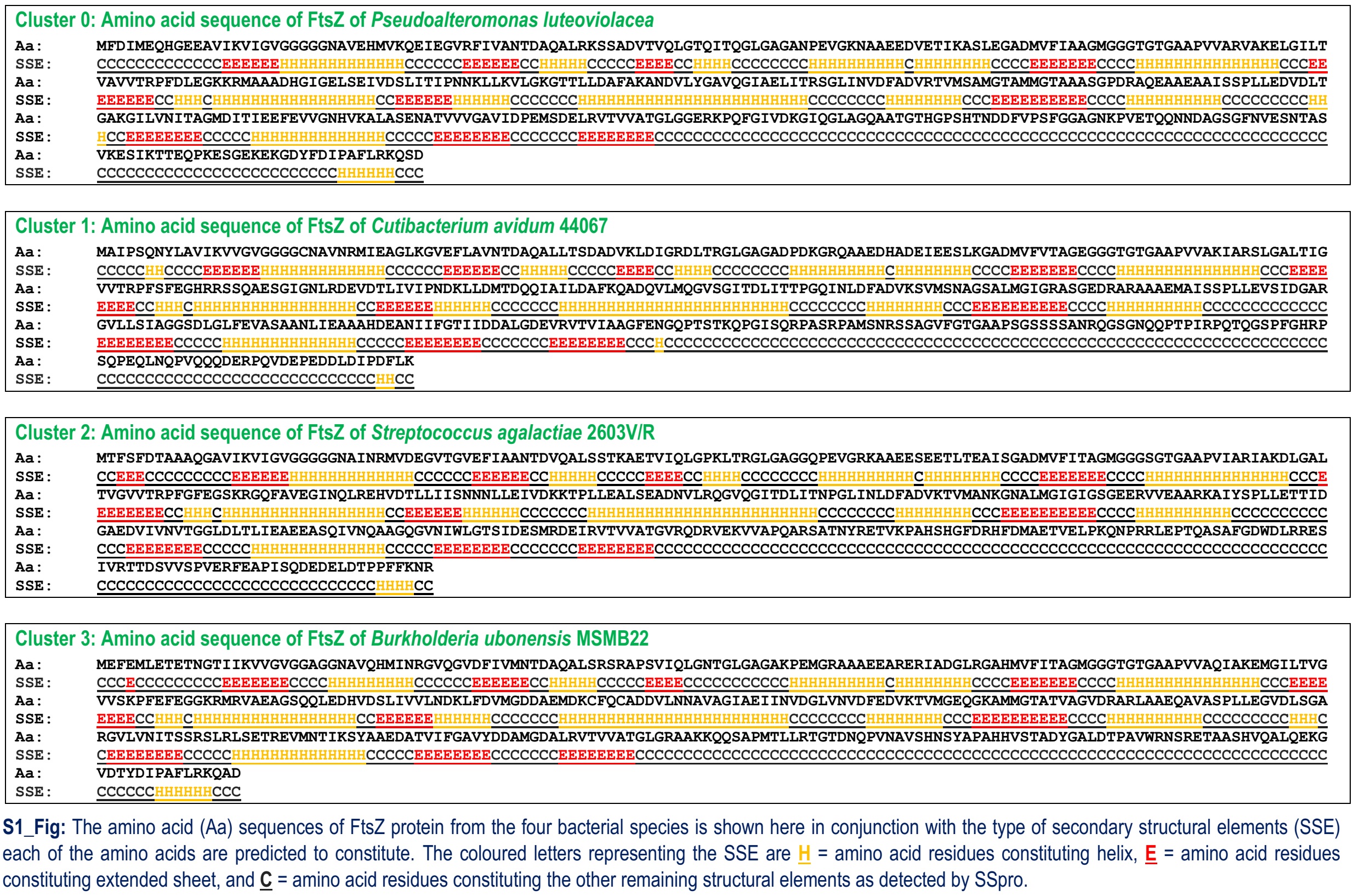

Supplement: S1 Fig — (JPG) [file pone.0219231.s010.jpg]

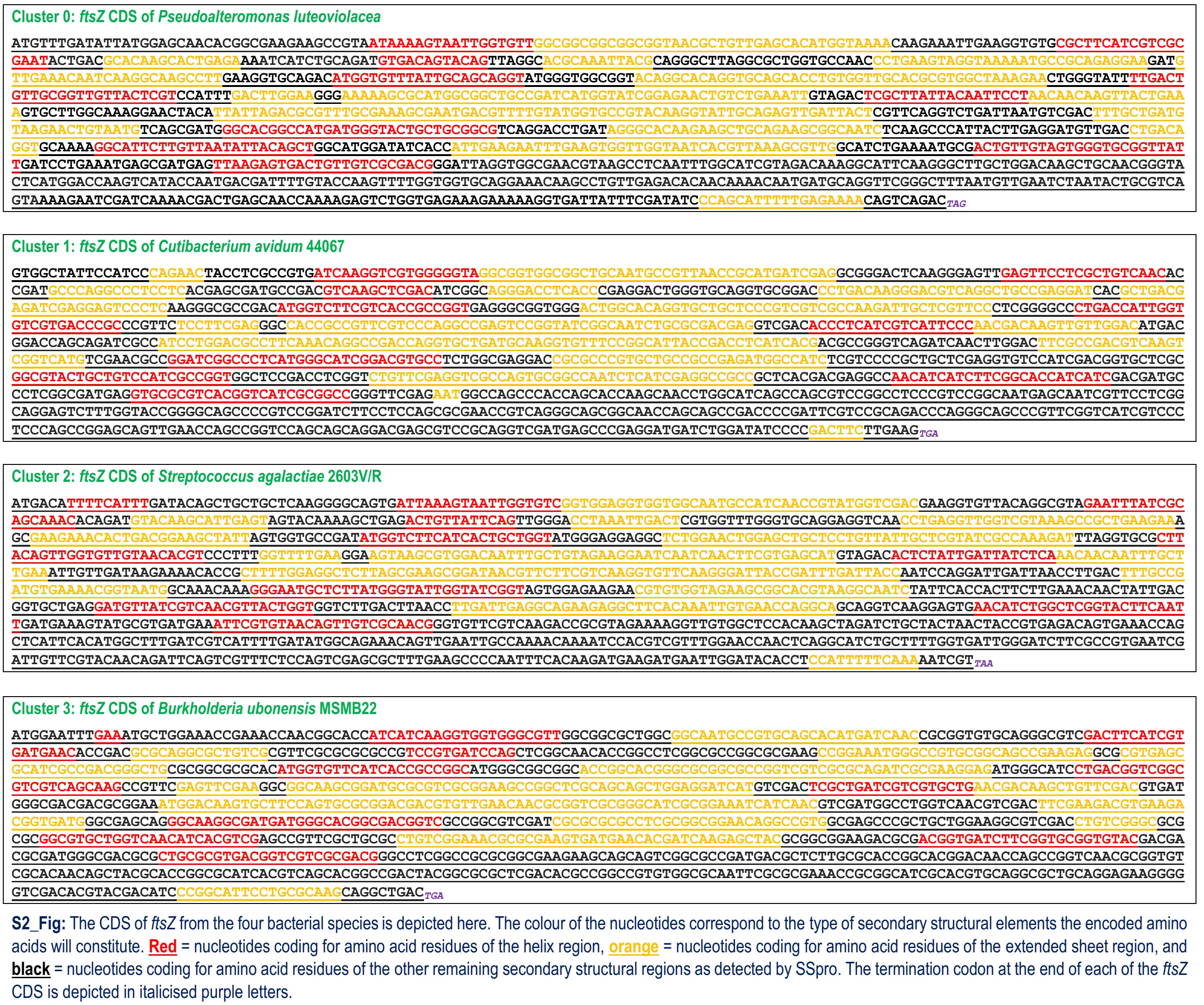

Supplement: S2 Fig — (JPG) [file pone.0219231.s011.jpg]
